# Extracellular vesicles derived from T regulatory cells suppress T cell proliferation and prolong allograft survival

**DOI:** 10.1038/s41598-017-08617-3

**Published:** 2017-09-14

**Authors:** Sistiana Aiello, Federica Rocchetta, Lorena Longaretti, Silvia Faravelli, Marta Todeschini, Linda Cassis, Francesca Pezzuto, Susanna Tomasoni, Nadia Azzollini, Marilena Mister, Caterina Mele, Sara Conti, Matteo Breno, Giuseppe Remuzzi, Marina Noris, Ariela Benigni

**Affiliations:** 1 IRCCS-Istituto di Ricerche Farmacologiche Mario Negri, Centro Ricerche Trapianti Chiara Cucchi de Alessandri e Gilberto Crespi Ranica, Bergamo, Italy; 2 Institut Hospital del Mar d’Investigacions Mèdiques, Barcelona, Spain; 3 Unit of Nephrology and Dialyisis Azienda Socio Sanitaria Territoriale (ASST) Papa Giovanni XXIII, Bergamo, Italy; 4 Unit of Nephrology and Dialyisis, Department of Biomedical and Clinical Sciences, University of Milan, Milan, Italy

## Abstract

We have previously shown that rat allogeneic DC, made immature by adenoviral gene transfer of the dominant negative form of IKK2, gave rise *in-vitro* to a unique population of CD4^+^CD25^−^ regulatory T cells (dnIKK2-Treg). These cells inhibited Tcell response *in-vitro*, without needing cell-to-cell contact, and induced kidney allograft survival prolongation *in-vivo*. Deep insight into the mechanisms behind dnIKK2-Treg-induced suppression of Tcell proliferation remained elusive. Here we document that dnIKK2-Treg release extracellular vesicles (EV) riched in exosomes, fully accounting for the cell-contact independent immunosuppressive activity of parent cells. DnIKK2-Treg-EV contain a unique molecular cargo of specific miRNAs and iNOS, which, once delivered into target cells, blocked cell cycle progression and induced apoptosis. DnIKK2-Treg-EV-exposed T cells were in turn converted into regulatory cells. Notably, when administered *in-vivo*, dnIKK2-Treg-EV prolonged kidney allograft survival. DnIKK2-Treg-derived EV could be a tool for manipulating the immune system and for discovering novel potential immunosuppressive molecules in the context of allotransplantation.

## Introduction

Organ transplantation is the most effective treatment for end-stage organ failure, and in recent decades immunosuppressive drugs have greatly increased graft survival in transplant patients^1^. However, since long-lasting immunosuppression can increase the occurrence of opportunistic infections and the incidence of cancer, one major aim of transplantation research is to find new ways to induce allograft acceptance/tolerance, eliminating or reducing the use of immunomodulatory molecules^2^.

In the past two decades, the tolerogenic/regulatory capacity of immature dendritic cells (DC) has been exploited to promote long-term allograft acceptance^3^. Administering immature donor or donor-antigen-loaded recipient DC, by themselves or through interaction with host DC, promotes the deletion of donor-reactive T cells and increases the number of donor-specific regulatory T cells (Treg)^3^. As an alternative approach, immature DC have been used to generate Treg *in-vitro* ^4, 5^. In this context, we showed that rat allogeneic DC made immature by blocking NF-kB through adenoviral gene transfer of the dominant negative form of IKK2 (dnIKK2), gave rise *in-vitro* to a unique population of CD4^+^CD25^−^ regulatory T cells (dnIKK2-Treg)^6, 7^. These cells potently inhibited the T cell response *in-vitro*, without the need for cell-to-cell contact, and induced prolongation of kidney allograft survival *in-vivo*. However, deep insight into the mechanism(s) behind dnIKK2-Treg suppression of T cell proliferation remains elusive.

Several cell contact-independent mechanisms have been involved in the suppression of T cell activation by Treg, including deprivation of cytokines, such as IL-2, and the release of inhibitory factors, including IL-10, TGF-β, IL-35 and galectin-1^5, 8^. More recently, Treg have been shown to release extracellular vesicles (EV) and exosomes^9–13^, which deliver suppressive messages into target cells, allowing cell contact-independent inter-cellular communication. Exosomes are small membrane vesicles of endocytic origin riched in bioactive messengers (both proteins and RNAs), which can be delivered into target cells after fusing with their plasma membranes^14–16^. The cargo of bioactive messengers, which dictates the effect on target cells, is strictly dependent on the cell from which vesicles derive and on its physiological status^15^. Indeed, exosomes secreted by mature DC efficiently engage T cell activation *in-vitro* as well as *in-vivo* ^17, 18^, whereas exosomes released by immature DC promote tolerogenic immune responses both *in-vitro* ^15, 19^ and *in-vivo* ^20, 21^. Interestingly, a recent paper documented that naturally occurring Treg release exosomes that suppress pathogenic T helper 1 (Th1) cells in murine colitis through the transfer of microRNA (miRNA)^10^.

This study was designed to: 1) evaluate whether dnIKK2-Treg released EV; 2) investigate whether EV played a role in mediating the cell contact-independent immunoregulatory properties of dnIKK2-Treg *in-vitro*; 3) search for the mechanism underlying their immunomodulatory effect; and 4) verify whether dnIKK2-Treg-derived EV affect graft survival in a kidney allotransplant model.

We found that dnIKK2-Treg release EV riched in exosomes (dnIKK2-Treg-EV) that account fully for the T cell regulatory activity of dnIKK2-Treg *in-vitro*. In addition, dnIKK2-Treg-EV were capable of converting T cells into regulatory cells and prolonged kidney allograft survival *in-vivo*. The mechanism underlying the immune-modulating capacity of dnIKK2-Treg-EV is based on their unique molecular cargo, consisting of specific miRNAs and iNOS enzyme, which, once delivered into naïve T cells, block cell cycle progression and induce apoptosis.

## Results

### DnIKK2-Treg release EV riched in exosomes

**Figure 1:**
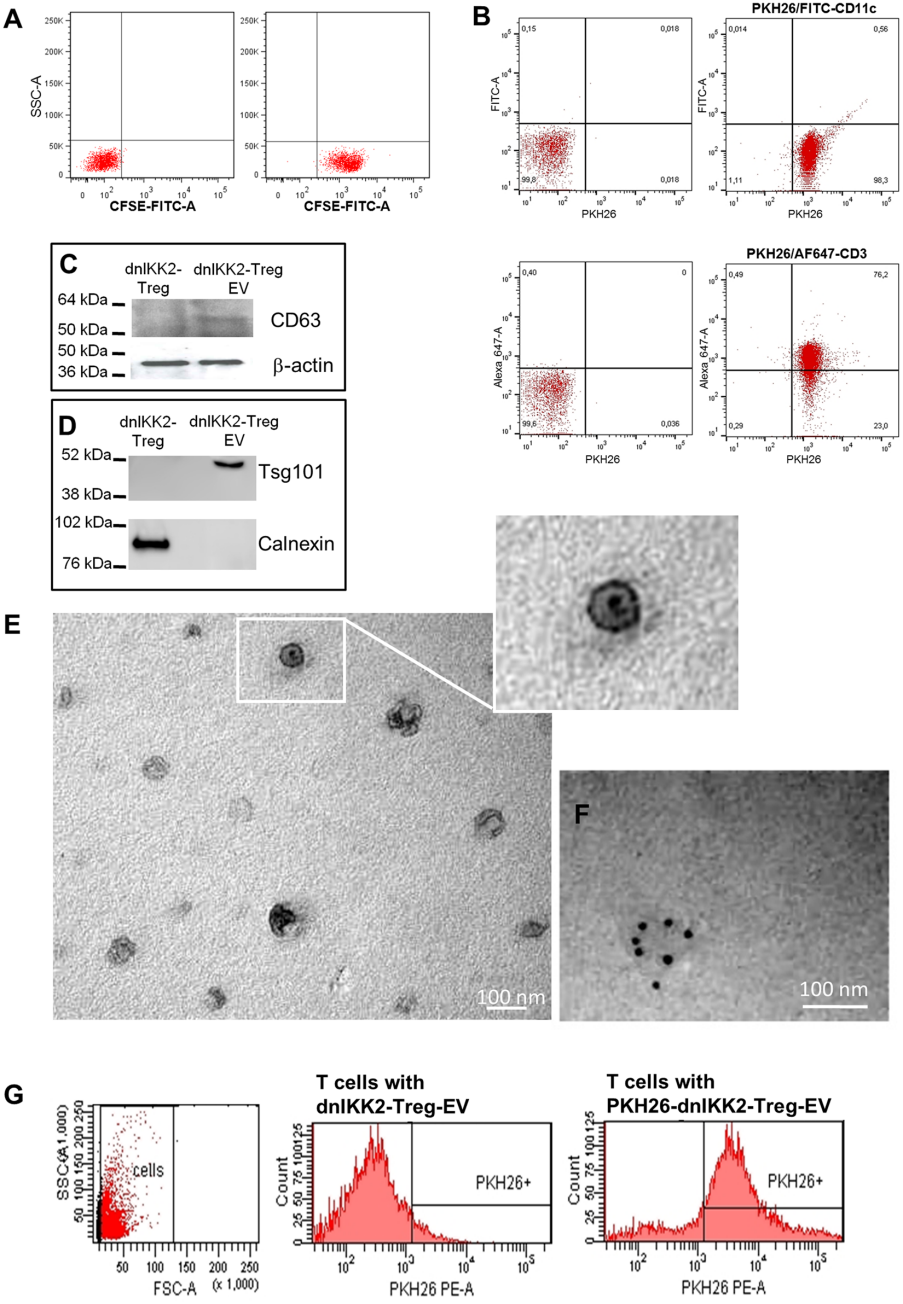
dnIKK2-Treg release extracellular vesicles riched in exosomes. (**A**) EV released by CFSE stained dnIKK2-Treg were CFSE^+^. DnIKK2-Treg were stained with CFSE, EV were prepared from conditioned medium, conjugated to latex beads and FACS-analyzed. Left dot plot: latex beads alone. Right dot plot: latex beads conjugated with dnIKK2-Treg-EV. One representative experiment of 3 is shown. (**B**) FACS analysis of membrane antigens on dnIKK2-Treg-EV. dnIKK2-Treg-EV were PKH26-labeled, conjugated to latex beads, stained with FITC-conjugated anti-CD11c antibody (upper panels), or AF647-conjugated anti-CD3 antibody (lower panels) and FACS-analyzed. Left dot plots: latex beads conjugated with not-stained dnIKK2-Treg-EV. Right dot plots: latex beads conjugated with PKH26-labelled and antibody-stained dnIKK2-Treg-EV. Analysis was done on gated beads, 5000 events are shown. One representative experiment of 3 is shown. (**C**) CD63 protein expression. CD63 in dnIKK2-Treg (left lane) or dnIKK2-Treg-EV (right lane) was analyzed by Western blot. For each lane 20 μg of total proteins were loaded. Upper panel: CD63 expression. Lower panel: β-actin expression. Blots were cropped. Molecular weights are given on the left. One representative experiment of 3 is shown. (**D**) Tsg101 and calnexin protein expression. Tsg101 and calnexin in dnIKK2-Treg (left lane) or dnIKK2-Treg-EV (right lane) were analyzed by Western blot. For each lane 15 μg of total proteins were loaded. Blots were cropped. Molecular weights are given on the left. One representative experiment of 3 is shown. (**E**) Electron Microscopy analysis of dnIKK2-Treg-EV. Representative micrograph displaying 40–100 nm vesicles with cup-shaped morphology in the 100,000 × g pellet obtained from conditioned medium of dnIKK2-Treg. Scale bar: 100 nm. (**F**) CD63 immunogold labeling. Electron micrograph of CD63 immunogold labeling. Scale bar: 100 nm. (**G**) Up-take of PKH26-stained dnIKK2-Treg-EV. FACS-analysis of PKH26-stained dnIKK2-Treg-EV-exposed Tcells. Left dot plot: gating strategy. Central histogram: cells exposed to unstained dnIKK2-Treg-EV. Right histogram: cells exposed to PKH26-stained dnIKK2-Treg-EV. One experiment of 2 is shown.

First we assessed the ability of dnIKK2-Treg to release EV. The majority of EV isolated from conditioned medium of CFSE-labeled dnIKK2-Treg were CFSE^+^ (Fig. 1A). PKH26-stained vesicles were negative for CD11c and positive for CD3 antigens (Fig. 1B), and these results confirmed that they were EV of T cell origin. The presence of CD63, as well as of Tsg101, a specific marker of vesicles of endocytic origin, and the absence of calnexin, a marker of vesicles of endoplasmic reticulum origin (Fig. 1C,D), suggested that dnIKK2-Treg-EV were mostly exosomes^22, 23^. Electron microscopy confirmed the nature of cup-shaped CD63^+^ exosomes, which measured 50–100 nm (Fig. 1E,F). Similarly to dnIKK2-Treg-EV, EV released from activated T cells (Tact-EV) expressed Tsg101 (Supplementary Fig. 1). Tsg101 expression was faint in EV from resting T cells (Trest-EV) indicating a low amount of vesicles of endocytic origin released from Trest (Supplementary Fig. 1). Both Tact-EV and Trest-EV were negative for calnexin (Supplementary Fig. 1).

### DnIKK2-Treg-EV suppress T cell proliferation

EV were taken up by target cells, as demonstrated by the fact that more than 75% of T cells expressed PKH26 after their exposure to PKH26-stained dnIKK2-Treg-EV (Fig. 1G). To test whether, following such engagement, dnIKK2-Treg-EV were responsible for the cell-to-cell contact-independent suppressive activity of dnIKK2-Treg, naïve T cell proliferation was evaluated at the end of a 4-day MLR in the presence of dnIKK2-Treg-EV.

**Figure 2:**
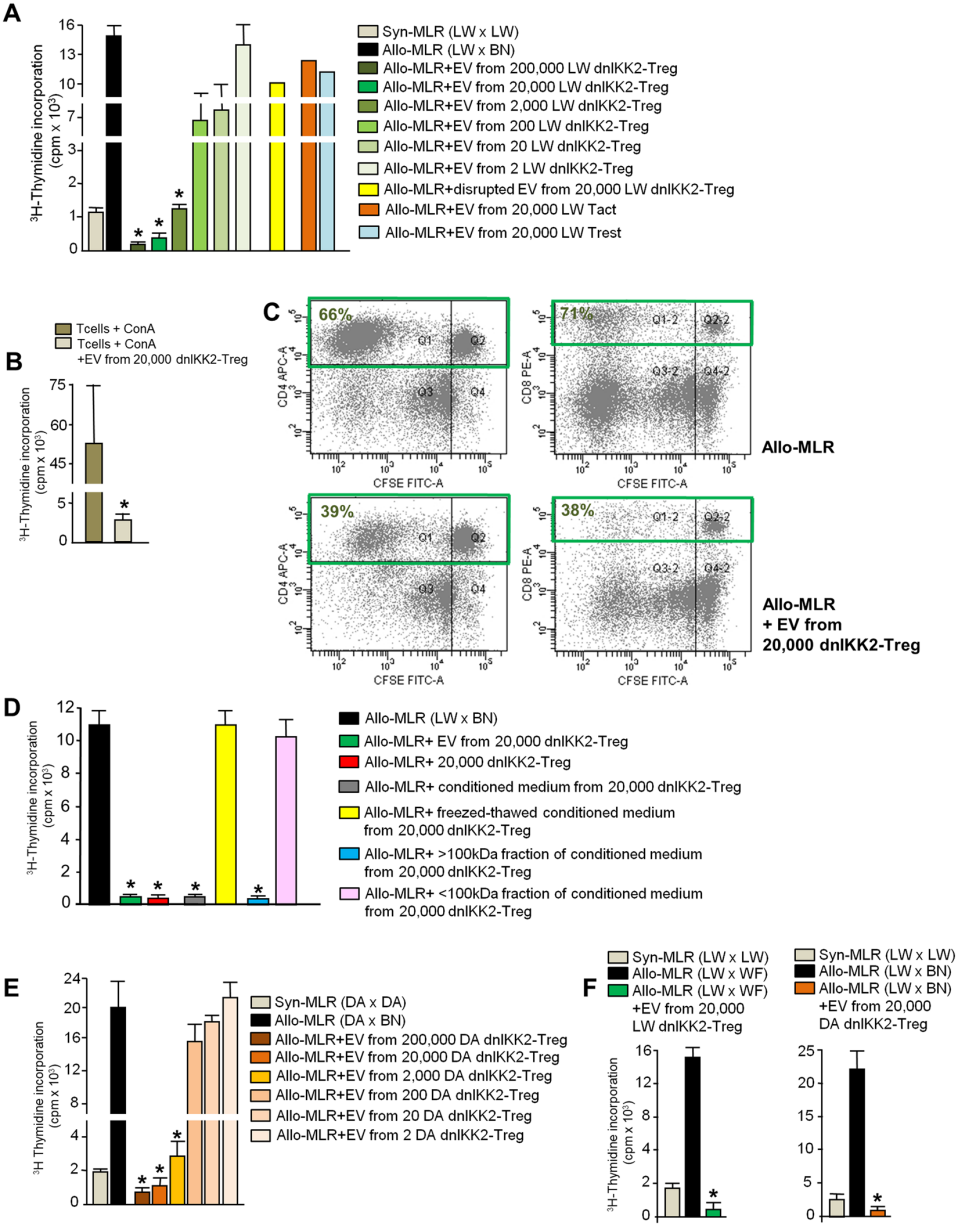
dnIKK2-Treg-EV suppress T cell proliferation. (**A**) Suppressive effect of LW dnIKK2-Treg-EV. A 4-day allogeneic MLR (Allo-MLR, 1 × 10^6^ LW lymph-node cells + 10,000 BN mature DC) was performed + /− EV from either dnIKK2-Treg (n = 5), or activated T cells (Tact, n = 2) or resting T cells (Trest, n = 2), or disrupted (freeze/thaw) dnIKK2-Treg-EV (n = 2). Syn-MLR: 1 × 10^6^LW lymph-node cells + 10,000 LW mature DC. Proliferation was measured by incorporation of ^3^H-Thymidine at day4 and expressed as cpm. Results are mean ± SE. *p < 0.05 vs all groups. (**B**) T cells were stimulated with ConA + /−dnIKK2-Treg-EV. Proliferation was measured by ^3^H-Thymidine incorporation and expressed as cpm (mean ± SD, n = 3, *p < 0.05 vs ConA). (**C**) A 4-day Allo-MLR was performed with (lower panels) or without (upper panels) EV from 20,000 dnIKK2-Treg. Left dot plots: double staining for CFSE and CD4. Right dot plots: double staining for CFSE and CD8. Percentages of CD4^+^ or CD8^+^ T cells that proliferated are given in upper left quadrants. One experiment of 3 is shown. (**D**). A 4-day Allo-MLR was performed + /−EV from 20,000 dnIKK2-Treg, or 20,000 dnIKK2-Treg, or conditioned medium from 20,000 dnIKK2-Treg (either untreated, or after 4–5 cycles of freeze and thaw, or after 100 kDa size exclusion filtering). Proliferation was measured by incorporation of ^3^H-Thymidine at day4 and expressed as cpm. (mean ± SD, n = 3, *p < 0.05 vs Allo-MLR, Allo-MLR + freezed-thawed conditioned medium, Allo-MLR + <100 kDa fraction of conditioned medium). (**E**) Suppressive effect of DA dnIKK2-Treg-EV. A 4-day Allo-MLR (1 × 10^6^ DA lymph-node cells + 10,000 BN mature DC) was performed + /−EV from DA dnIKK2-Treg. Proliferation was measured by incorporation of ^3^H-Thymidine at day4 and expressed as cpm. Results are mean ± SE. *p < 0.05 vs all groups (n = 3). (**F**) A 4-day Allo-MLR (1 × 10^6^ LW lymph node cells + 10,000 WF mature DC, left panel, or + 10,000 BN mature DC, right panel) was performed + /−LW (left panel) or DA (right panel) dnIKK2-Treg-EV. Proliferation was measured by incorporation of ^3^H-Thymidine at day4 and expressed as cpm. Results are mean ± SE. *p < 0.05 vs all groups (n = 3).

As shown in Fig. 2A, dnIKK2-Treg-EV potently suppressed T cell proliferation in allogeneic MLR (allo-MLR, LWxBN) in a dose-dependent manner. A significant inhibition of T cell proliferation was achieved by EV from 2,000 dnIKK2-Treg but was further increased in the presence of EV from 20,000 to 200,000. EV from 2 to 200 dnIKK2-Treg did not inhibit T cell proliferation. So we elected to use EV from 20,000 dnIKK2-Treg (corresponding to approximately 40–60 ng of proteins) for further experiments. The suppressive activity was lost completely when dnIKK2-Treg-EV were disrupted by 4–5 cycles of freeze and thaw (Fig. 2A). EV released by either CD4^+^activated (Tact-EV) or CD4^+^ resting T cells (Trest-EV) did not affect T cell proliferation (Fig. 2A). Notably, dnIKK2-Treg-EV were able to suppress even T cells stimulated by a polyclonal stimulus, such as Concanavalin A (Fig. 2B), suggesting that dnIKK2-Treg-EV exerted their suppressive activity directly on T cells and not through the inhibition of DC stimulatory capacity.

The anti-proliferative effect of dnIKK2-Treg-EV was confirmed by the CFSE dilution assay showing that CD3^+^ T cell proliferation was reduced by 43 ± 10% (mean ± SD, n = 3) at the end of MLR performed in the presence of EV from 20,000 dnIKK2-Treg, compared to an allo-MLR. A more detailed analysis showed that proliferation of both CD4^+^ and CD8^+^ T cells was reduced in the presence of dnIKK2-Treg-EV (Fig. 2C and Supplementary Fig. 2). The percentage of proliferation reduction was similar in the CD4^+^ and CD8^+^ T cell subsets (44 ± 13% and 41 ± 5% respectively, mean ± SD, n = 3).

DnIKK2-Treg-EV fully accounted for the suppressive effect originally described with the cells of origin^6, 7^, since a comparable suppressive effect was achieved either by EV from 20,000 dnIKK2-Treg or by 20,000 dnIKK2-Treg (Fig. 2D). To further confirm that dnIKK2-Treg-EV accounted for the suppressive effect, T cell proliferation was assessed with conditioned medium before and after size exclusion filtering. As expected, conditioned medium inhibited T cell proliferation and the activity was lost after freezing and thawing (Fig. 2D). The inhibiting activity was confined to the >100 kDa fraction that was enriched in EV while it was not observed with <100 kDa fraction of conditioned medium (Fig. 2D).

To rule out the possibility of dnIKK2-DC-derived EV potentially contaminating dnIKK2-Treg-EV, we prepared EV from dnIKK2-Treg obtained at the end of allogeneic MLR with LN cells from DA rats. Through this approach we were able to obtain EV from sorted RT1A^a+^CD4^+^ DA dnIKK2-Treg, without any contaminating BN dnIKK2-DCs. Results showing that DA dnIKK2-Treg-EV suppressed T cell proliferation like LW dnIKK2-Treg-EV did, allowed us to exclude the possibility that the suppressive capacity of dnIKK2-Treg-EV was due to contaminating EV of dnIKK2-DC origin (Fig. 2E).

DnIKK2-Treg-EV did not show alloantigen-specificity, indeed they suppressed T cell proliferation even toward third party allogeneic WF DCs (Fig. 2F, left panel). In addition EV from DA dnIKK2-Treg suppressed proliferation of non autologous LW T cells toward BN DCs (Fig. 2F, right panel).

### Suppression of T cell alloreactivity induced by dnIKK2-Treg-EV has latency period

**Figure 3:**
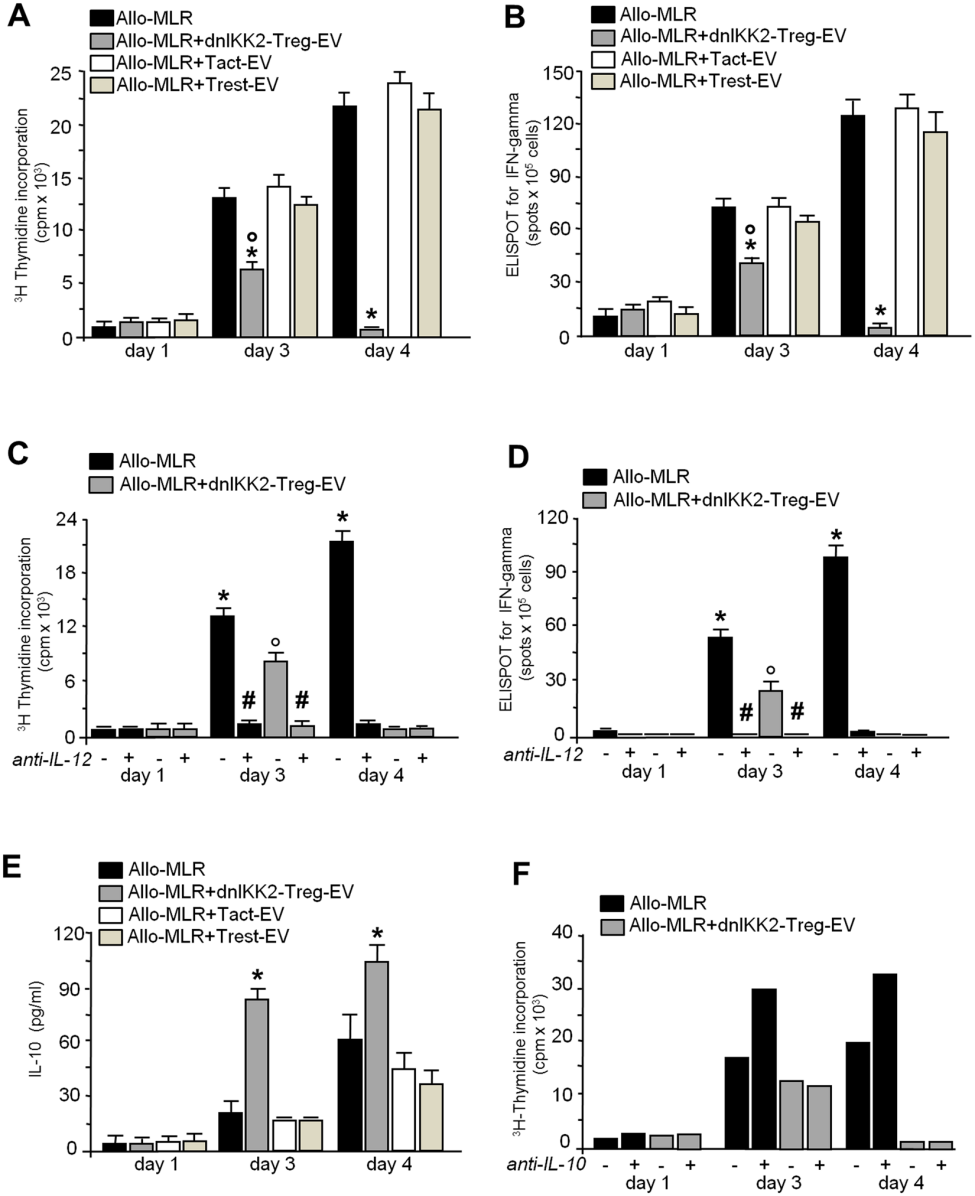
Suppressive effect of dnIKK2-Treg-EV on T cell proliferation and activation has a latency period. (**A,B**). A 4-day allogeneic MLR (Allo-MLR, 1 × 10^6^ LW lymph node cells plus 10,000 BN mature DC) was performed in the presence or absence of EV from 20,000 dnIKK2-Treg (dnIKK2-Treg-EV), or 20,000 activated T cells (Tact-EV) or 20,000 resting T cells (Trest-EV). At day 1, 3 and 4 of MLR, proliferation was measured by incorporation of ^3^H-Thymidine and expressed as cpm (**A**); frequency of IFNγ-producing T cells was assessed by ELISPOT assay (**B**). Results are mean ± SE of 5 independent experiments. *p < 0.05 vs all groups at the same time point. °p < 0.05 vs naïve MLR + dnIKK2-Treg-EV at day 1. (**C,D**). A 4-day Allo-MLR was performed in the presence or absence of EV from 20,000 dnIKK2-Treg (dnIKK2-Treg-EV) + /- anti-IL-12 antibody. At day 1, 3 and 4 of MLR, proliferation was measured by incorporation of ^3^H-Thymidine and expressed as cpm (**C**); frequency of IFNγ-producing T cells was assessed by ELISPOT assay (**D**). Results are mean ± SE of 3 independent experiments. *p < 0.05 vs all groups at the same time point. °p < 0.05 vs naïve MLR + dnIKK2-Treg-EV at day 1. ^#^p < 0.05 vs without anti-IL-12. (**E**) A 4-day Allo-MLR was performed in the presence or absence of EV from 20,000 dnIKK2-Treg (dnIKK2-Treg-EV), or 20,000 activated T cells (Tact-EV) or 20,000 resting T cells (Trest-EV). IL-10 release was measured in the MLR supernatant at day1, 3 and 4 by ELISA assay. Results are expressed as mean ± SE of 5 independent experiments. *p < 0.05 vs all groups at the same time point. (**F**) A 4-day Allo-MLR was performed in the presence or absence of EV from 20,000 dnIKK2-Treg (dnIKK2-Treg-EV) + /− anti-IL-10 antibody. Proliferation was measured by incorporation of ^3^H-Thymidine at day1, 3 and 4 of MLR and expressed as cpm. Results are mean of 2 independent experiments.

A time course of T cell alloreactivity with dnIKK2-Treg-EV in allo-MLR is shown in Fig. 3. At day 3, dnIKK2-Treg-EV inhibited T cell proliferation compared to naïve MLR, while Tact or Trest-EV did not affect T cell proliferation. At day4, dnIKK2-Treg-EV completely suppressed T cell proliferation, which on the other hand further increased in all the control MLRs (Fig. 3A).

In line with the results of T cell proliferation and according to results obtained with EV from CD4^+^CD25^+^Foxp3^+^Treg^11^, the evaluation of IFN-γ^+^ clone generation showed that at day 3 dnIKK2-Treg-EV inhibited the formation of clones of IFN-γ producing cells. At day 4, IFN-γ^+^ clones were almost undetectable when T cells were stimulated by allogeneic DCs in the presence of dnIKK2-Treg-EV, whereas they further rose in all the control MLRs (Fig. 3B). Evidence that IFN-γ producing cells were CD4^+^Th1/CD8^+^Tc1 cells emerged from experiments showing that the addition of a neutralizing anti-IL-12 antibody, the master cytokine for Th1/Tc1 clone formation, completely blocked T cell proliferation (Fig. 3C) and erased the number of IFN-γ^+^ cell clones (Fig. 3D), both in MLR added with dnIKK2-Treg-EV and in allo-MLR.

A higher amount of IL-10 was found in the supernatant of MLR performed with dnIKK2-Treg-EV compared to all the control MLR conditions (Fig. 3E). However, the addition of an anti-IL-10 antibody did not restore T cell proliferation (Fig. 3F), indicating that the anti-proliferative effect of dnIKK2-Treg-EV was not mediated by IL-10.

### dnIKK2-Treg-EV prevent cell cycle progression of quiescent T cells and induce apoptosis

**Figure 4:**
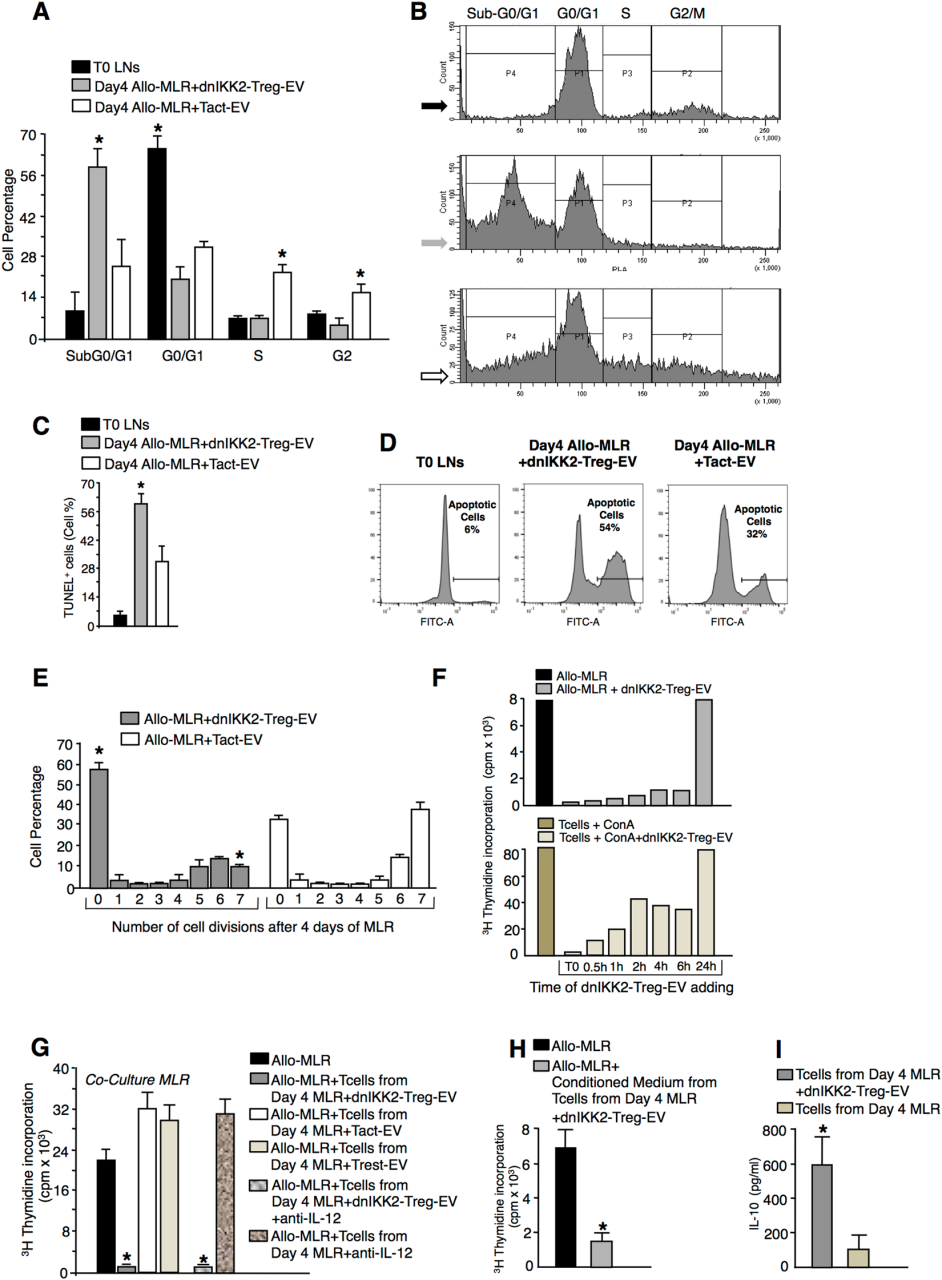
Effect of dnIKK2-Treg-EV on cell cycle progression and apoptosis. (**A,B**) Cell cycle distribution in dnIKK2-Treg-EV-exposed Tcells. A 4-day Allo-MLR (1 × 10^6^ LW lymph-node cells + 10,000 BN mature DC) was performed with dnIKK2-Treg-EV or Tact-EV. (**A**): percentages of T cells in sub-G0/G1, G0/G1, S and G2/M phases. Mean ± SD (n = 4 independent experiments). *p < 0.05 vs all groups in the same cell cycle phase. (**B**): Representative FACS histograms of PI staining and gates at day 0 (upper histogram) or 4 of MLR (+dnIKK2-Treg-EV: central histogram; +Tact-EV: lower histogram). (**C–D**) Apoptosis in dnIKK2-Treg-EV-exposed Tcells. A 4-day Allo-MLR was performed with dnIKK2-Treg-EV or Tact-EV. (**C**): Percentages of TUNEL^+^ cells (by FACS). Mean ± SD (n = 3 independent experiments). *p < 0.05 vs day4 Allo-MLR + Tact-EV. (**D**): Representative FACS histograms. (**E**) Numbers of cell divisions in dnIKK2-Treg-EV-exposed Tcells. A 4-day Allo-MLR was performed with dnIKK2-Treg-EV or Tact-EV. Cell division number was evaluated by FlowJo software after FACS-analysis of CFSE-labeling on 7-AAD^-^ cells. Mean ± SD (n = 4 independent experiments). *p < 0.05 vs corresponding Allo-MLR + Tact-EV. (**F**) dnIKK2-Treg-EV suppress cell proliferation only when given early during T cell stimulation. T cells were stimulated with allogeneic DC (Allo-MLR, upper panel) or with ConA (lower panel), + /− dnIKK2-Treg-EV, administered at the beginning of stimulation (T0) or after 0.5–24 h. Proliferation was measured at the end of stimulation by ^3^H-Thymidine incorporation and expressed as cpm. Mean of 2 independent experiments. (**G**) dnIKK2-Treg-EV-exposed Tcells acquire Treg capacity. A co-culture MLR was performed with 10,000 T cells, harvested at the end of MLR carried out with dnIKK2-Treg-EV, or Tact-EV or Trest-EV, and added to an Allo-MLR. Co-culture MLR was also performed with T cells harvested at the end of MLR + dnIKK2-Treg-EV + anti-IL12 or MLR + anti-IL12. Proliferation was measured by ^3^H-Thymidine incorporation and expressed as cpm. Results Mean ± SE (n = 5 independent experiments). *p < 0.05 vs all groups. (**H**) Conditioned medium from 20,000 T cells harvested from day4 allo-MLR + dnIKK2-Treg-EV was added or not to Allo-MLR. Proliferation was expressed as cpm. Mean ± SD, n = 3, *p < 0.05vs Allo-MLR. (**I**) T cells were harvested from day4 allo-MLR + /−dnIKK2-Treg-EV and IL-10 release was measured (by ELISA) after 16 h stimulation by allogeneic DC. Mean ± SD, n = 3, *p < 0.05 vs Tcells from day4 MLR.

Next we tested whether dnIKK2-Treg-EV blocked the cell cycle progression of T cells in MLR. T cells in the G0/G1 cell cycle phase were stimulated by allogeneic DC in the presence of dnIKK2-Treg-EV. After 4 days of MLR, T cells did not progress toward the S and G2 phases (Fig. 4A,B) but most of them (55 ± 8%) underwent apoptosis (subG1). In contrast, T cells stimulated by allogeneic DC in the presence of Tact-EV were in S (19 ± 4%) and G2/M (14 ± 5%) phases (p < 0.05 vs dnIKK2-Treg-EV-exposed T cells), and the percentage of cells in subG1 was significantly lower (p < 0.05) than that of T cells exposed to dnIKK2-Treg-EV (Fig. 4A,B). Consistently, the percentage of apoptotic T cells, as measured using TUNEL (Fig. 4C,D) or AnnexinV/7AAD staining (Supplementary Fig. 3), was higher after 4 days of MLR with dnIKK2-Treg-EV compared to allo-MLR + Tact-EV. Western blot results showing a lack of FasL, granzyme B, perforin or galectin 9 in protein extracts from dnIKK2-Treg-EV suggested that apoptosis was not dependent on these pro-apoptotic molecules (Supplementary Fig. 4). Cell cycle progression of T cells in allo-MLR was not influenced by Tact-EV, as shown by comparable cell cycle distribution at day 4 of MLR with or without Tact-EV (Supplementary Fig. 5).

The evaluation of cell division numbers revealed that the majority of T cells stimulated by allogeneic DC in the presence of dnIKK2-Treg-EV did not undergo cell division (Fig. 4E). Only 10% of dnIKK2-Treg-EV exposed T cells completed 7 rounds of division in contrast with 35% of T cells exposed to Tact-EV. Suppressive activity by dnIKK2-Treg-EV was exerted when they were added within 6 hours of T cell stimulation, while it was lost when the addition occurred after 24 h (Fig. 4F), indicating that dnIKK2-Treg-EV cannot exert suppressive activity on already proliferating/activated T cells. Similar results were obtained when target T cells were stimulated by ConA (Fig. 4F).

### DnIKK2-Treg-EV convert naïve T cells into Treg

Naïve T cells, exposed to dnIKK2-Treg-EV during MLR, not only underwent a forced block in cell cycle progression but also a functional change. Indeed, T cells recovered from MLR with dnIKK2-Treg-EV and co-cultured onto an allo-MLR (co-culture MLR), inhibited naïve T cell proliferation toward allogeneic DC, suggesting that dnIKK2-Treg-EV had converted them into Treg (Fig. 4G). In contrast, T cells harvested from MLR performed in the presence of either Tact-EV or Trest-EV did not influence T cell proliferation in a co-culture MLR (Fig. 4G). T cells exposed to dnIKK2-Treg-EV did not express CD25 and FoxP3 (1.7 ± 0.7% of CD25^+^FoxP3^+^on CD3^+^CD4^+^ dnIKK2-Treg-EV-exposed T cells vs 5.7 ± 1.0% of CD25^+^FoxP3^+^ on CD3^+^CD4^+^ naive T cells, n = 3, by FACS analysis, p = NS, Supplementary Fig. 6A) and did not need cell-to-cell contact with target T cells to exert regulatory activity that was fully mirrored by their conditioned medium (Fig. 4H). A greater amount of IL-10 was released from T cells exposed to dnIKK2-Treg-EV (Fig. 4I) as compared to T cells isolated from MLR performed in the absence of dnIKK2-Treg-EV. Real time PCR experiments, showed no difference in the expression of CD49b and LAG3 in T cells exposed to dnIKK2-Treg-EV during MLR as compared to T cells in MLR alone (Supplementary Fig. 6B). By FACS-analysis the percentage of CD3^+^CD4^+^ T cells co-expressing CD49b and LAG3 did not differ between T cells exposed to dnIKK2-Treg-EV during MLR and T cells in MLR alone (Supplementary Fig. 6C).

Additional real time PCR analysis, documented that both T cells activated in MLR in the presence of dnIKK2-Treg-EV and T cells not-exposed to dnIKK2-Treg-EV during MLR, expressed lower levels of PD-1 and higher levels of CTLA-4 and Tim3 than naïve T cells at day0 allo-MLR (Supplementary Fig. 7A). However, Tim3 mRNA expression and the percentage of CD3^+^Tim3^+^ T cells were significantly higher in T cells exposed to dnIKK2-Treg-EV during MLR than in not exposed (day0 allo-MLR, 5.4 ± 3.9% of Tim3^+^ cells on CD3^+^ T cells; day4 allo-MLR, 37.6 ± 16.1% of Tim3^+^ cells on CD3^+^ T cells; day4 allo-MLR + dnIKK2-Treg-EV, 74.8 ± 3.6% of Tim3^+^ cells on CD3^+^ T cells; n = 3, Supplementary Fig. 7B,C).

The emergence of Treg from MLR with dnIKK2-Treg-EV was not due to the block of Th1/Tc1, as T cells harvested at the end of a control allogeneic MLR carried out in the presence of anti-IL-12 antibody (to prevent the emergence of Th1/Tc1) did not suppress an MLR in co-culture (Fig. 4G).

### DnIKK2-Treg-EV contain a subset of specific miRNA

**Figure 5:**
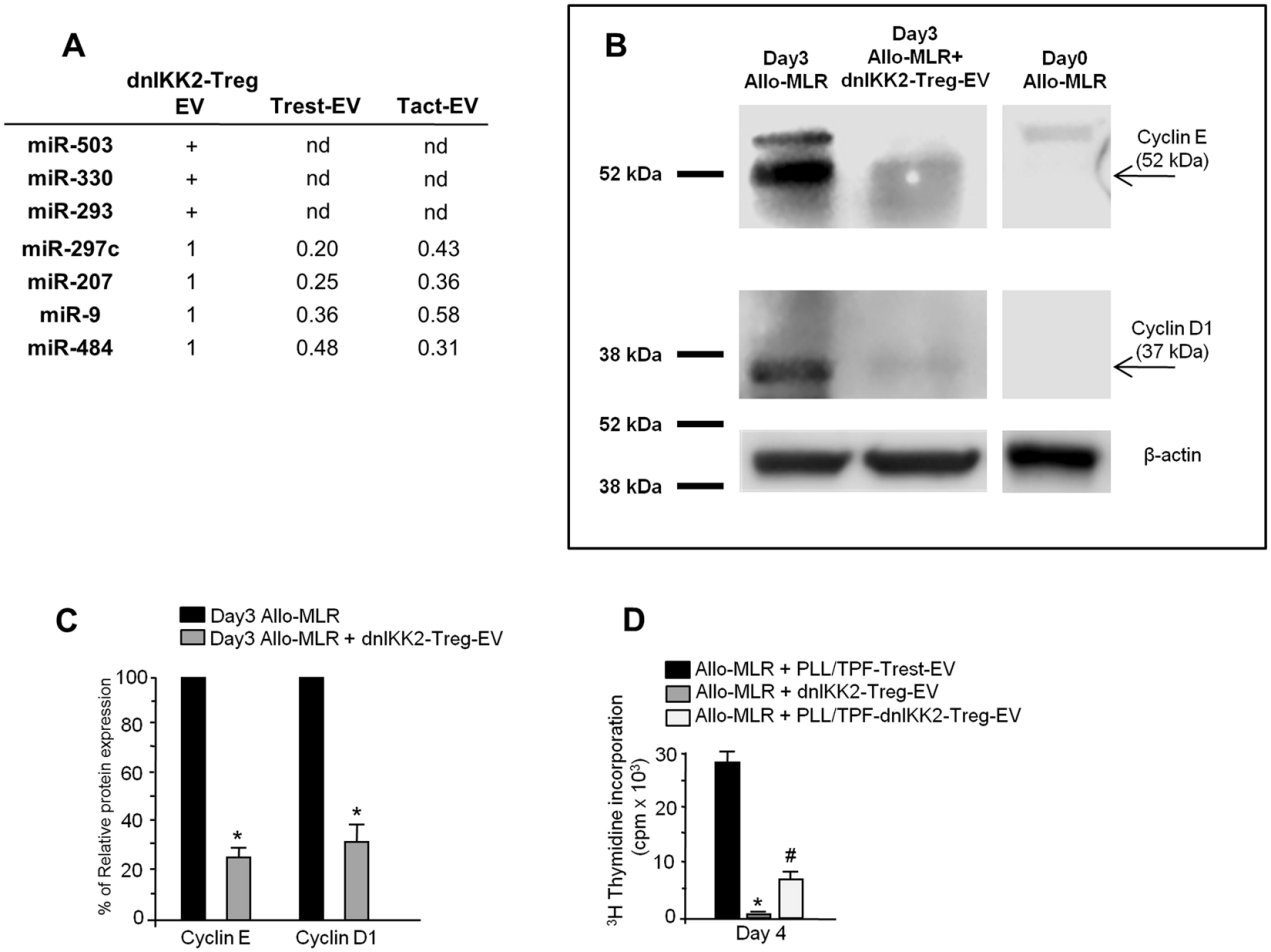
dnIKK2-Treg-EV contain a specific subset of miRNAs. (**A**) miRNAs expressed in dnIKK2-Treg-EV. We used the ΔΔCt technique, when possible, to calculate cDNA content, using dnIKK2-Treg-EV cDNA expression as calibrator. The expression of the housekeeping snRNA U6 was comparable among dnIKK2-Treg-EV, Tact-EV and Trest-EV (average Ct values: 26.55, 27.56 and 27.40 respectively). + : detected, nd: not detectable. (**B,C**) Expression and quantification of Cyclin E and Cyclin D1 in cells from allogeneic MLR exposed to dnIKK2-Treg-EV. (**B**)Western blot analysis of Cyclin E, Cyclin D1 and β-actin in protein extracts of cells from day 0 MLR (right lane) or day 3 MLR performed with (central lane) or without (left lane) dnIKK2-Treg-EV. For each lane 20 μg of total proteins were loaded. Blots were cropped. Molecular weights are given on the left. One representative experiment of 3 is shown. (**C**) results of densitometric analysis are given, after normalization with β-actin, as % of relative expression, considering MLR performed without dnIKK2-Treg-EV as 100%. Mean ± SD of 3 independent experiments.*p < 0.05 vs corresponding Allo-MLR. (**D**) Effect of PLL/TPF-dnIKK2-Treg-EV on T cell proliferation. An allogeneic MLR (Allo-MLR, 1 × 10^6^ LW lymph node cells plus 10,000 BN mature DC) was performed in the presence of EV from 20,000 Trest treated with poly-L-lysine (PLL) and trypaflavine (TPF) (PLL/TPF-Trest-EV, used as controls), or from 20,000 dnIKK2-Treg (dnIKK2-Treg-EV), or from 20,000 dnIKK2-Treg treated with PLL/TPF (PLL/TPF-dnIKK2-Treg-EV). Proliferation was measured by incorporation of ^3^H-Thymidine at day 4 of MLR and expressed as cpm. Results are expressed as mean ± SE of 3 independent experiments. *p < 0.05 vs all groups. ^#^p < 0.05 vs Allo-MLR + PLL/TPF-Trest-EV.

EV can act as an intercellular shuttle of RNA and miRNAs. To assess whether dnIKK2-Treg-EV exert their suppressive function through miRNAs, their profile was assessed in dnIKK2-Treg-EV, Tact-EV and Trest-EV. A total of 87 miRNAs was detected, of which 56 were exclusively expressed in dnIKK2-Treg-EV (Supplementary Tables 1 and 2). Of the latter, 9 were highly expressed with threshold cycle (Ct) values below 30. RT-PCR validation of these 9 miRNAs confirmed the high expression of 7 miRNAs, of which 3 (miR-503, miR-330 and miR-293) were exclusively expressed in dnIKK2-Treg-EV, while the remaining 4 miRNAs (miR-297c, miR-207, miR-9, miR-484) were faintly detected in Tact-EV and Trest-EV too (Fig. 5A).

Target predictions and pathway analysis for the 7 miRNAs were performed with miRPath software^24^ based on gene-miRNA interactions validated in humans. This analysis highlighted 3 miRNAs (miR-503, miR-330 and miR-9) potentially affecting 16 pathways (Supplementary Table 3) among which cell cycle was the most closely related to anti-proliferative effects of dnIKK2-Treg-EV. Target prediction of cell cycle genes targeted by miR-503, miR-330 and miR-9 included CCNE1, CCNE2, CCND1, CDC14A, E2F1-3, CDKN1A, CDC25A, CHEK1, WEE1 and EP300 (Supplementary Fig. 8)^25, 26^. Notably, the expression of Cyclin E and Cyclin D1, two predicted targets crucial for G1 phase execution and G1/S progression, was lower in T cells exposed to dnIKK2-Treg-EV during MLR compared to T cells activated in the absence of dnIKK2-Treg-EV (Fig. 5B,C). Tact-EV did not influence expression of Cyclin E and Cyclin D1 in T cells stimulated by allogeneic DC in MLR (Supplementary Fig. 9).

To investigate the role of the miRNAs in the suppressive activity of dnIKK2-Treg-EV, EV of dnIKK2-Treg were obtained in the presence of poly-L-lysine (PLL) and trypaflavine (TPF), small molecules affecting miRNA generation and stability^27, 28^. DnIKK2-Treg-EV inhibition of T cell proliferation, evaluated at day 4 of MLR, was not completely restored by PLL/TPF (Fig. 5D). Specificity of miRNA inhibition by PLL/TPF treatment was documented by results of RT-PCR showing that miR503 was undetectable (Ct values > 40) in EV released from PLL/TPF-treated dnIKK2-Treg, at variance with EV from untreated dnIKK2-Treg (Ct values < 35).

### DnIKK2-Treg-EV contain iNOS mRNA and protein

**Figure 6:**
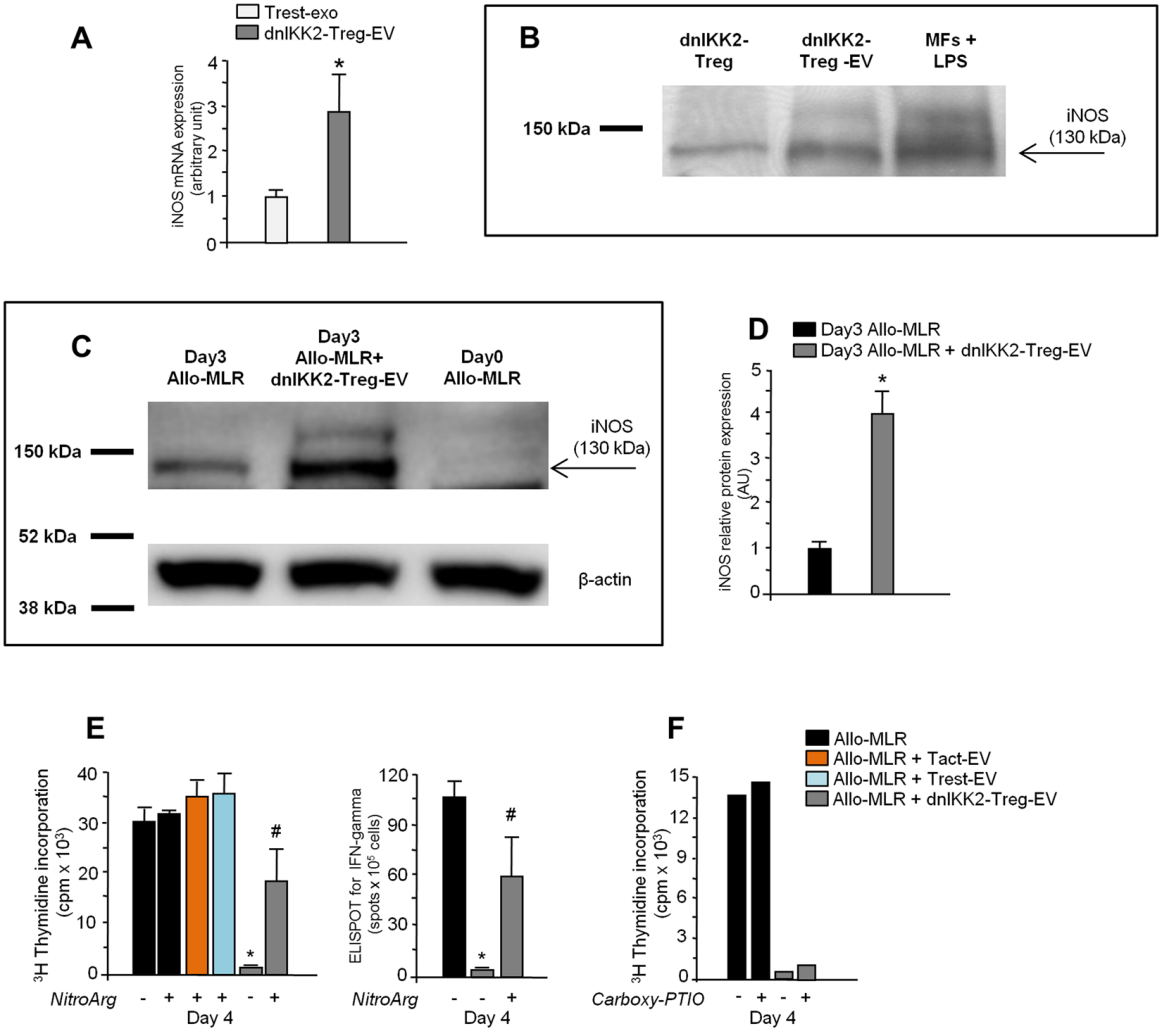
dnIKK2-Treg-EV contain iNOS. (**A**) iNOS mRNA in dnIKK2-Treg-EV. Inducible NOS (iNOS) mRNA analysis in dnIKK2-Treg-EV and Trest-EV by real-time PCR. The cDNA content was calculated by ΔΔCt technique, using as calibrator the cDNA expression in Trest-EV. Results are expressed as arbitrary units (AUs), mean ± SD (n = 3 independent experiments). *p < 0.05 vs Trest-EV. (**B**) iNOS protein expression. iNOS protein expression in dnIKK2-Treg (left lane), or dnIKK2-Treg-EV (central lane), or LPS-stimulated rat peritoneal macrophages (MFs) as positive control (right lane), by Western blot. For each lane 20 μg of total proteins were loaded. Blot was cropped. Molecular weights are given on the left. One representative experiment of 3 is shown. (**C,D**) Expression and quantification of iNOS protein in allogeneic MLR performed in the presence of dnIKK2-Treg-EV. (**C**) iNOS protein expression (by Western blot) in protein extracts of cells from day 0 MLR (right lane) or day 3 MLR with (central lane) or without (left lane) dnIKK2-Treg-EV. For each lane 19 μg of total proteins were loaded. Blots were cropped. Molecular weights are given on the left. One representative experiment of 3 is shown. (**D**) results of densitometric analysis are given, after normalization with β-actin, as arbitrary unit (AU) considering naïve MLR as 1. Mean ± SD (n = 3 independent experiments).*p < 0.05 vs naïve Allo-MLR. (**E**) Effect of NOS inhibition on cell proliferation and IFN-γ^+^ clone generation in dnIKK2-Treg-EV-exposed T cells. A 4-day Allo-MLR (1 × 10^6^ LW lymph-node cells + 10,000 BN mature DC) was performed with or without EV from 20,000 dnIKK2-Treg (dnIKK2-Treg-EV) or Tact (Tact-EV) or Trest (Trest-EV) and + /− N-ω-nitro-L-arginine (NitroArg). Proliferation was measured by ^3^H-Thymidine incorporation at day4 and expressed as cpm (left panel); frequency of IFNγ-producing T cells was assessed by ELISPOT (right panel). Results are expressed as mean ± SE (n = 3 independent experiments). *p < 0.05 vs all groups. ^#^p < 0.05 vs ctr Allo-MLRs. (**F**) Effect of Carboxy-PTIO on cell proliferation in dnIKK2-Treg-EV-exposed T cells. A 4-day Allo-MLR was performed with or without EV from 20,000 dnIKK2-Treg (dnIKK2-Treg-EV) and + /− Carboxy-PTIO (5 μM). Proliferation was measured by ^3^H-Thymidine incorporation at day4 and expressed as cpm.

Since miRNA cargo did not fully account for the anti-proliferative effect of dnIKK2-Treg-EV and on the basis of our previously reported data on iNOS mRNA and protein expression in dnIKK2-Treg^6^, we further assessed whether iNOS was shuttled in dnIKK2-Treg-EV. Real-time PCR showed that iNOS mRNA was present within dnIKK2-Treg-EV at a significantly higher level than that found in control Trest-EV (Fig. 6A). Consistently, Western blot analysis revealed a strong specific signal for iNOS in protein extracts from dnIKK2-Treg-EV, which was higher than that of dnIKK2-Treg (Fig. 6B), suggesting selective transfer of iNOS protein in EV. In addition, Western blot analysis revealed that cells exposed to dnIKK2-Treg-EV during MLR displayed a 4-fold higher iNOS expression than that recorded in allo-MLR (Fig. 6C,D), indicating that iNOS mRNA and protein were transferred from dnIKK2-Treg-EV to T cells. Tact-EV did not influence expression of iNOS in T cells stimulated by allogeneic DC in MLR (Supplementary Fig. 9).

To investigate the involvement of iNOS in the suppressive effect of dnIKK2-Treg-EV, MLR experiments were repeated in the presence of the NOS inhibitor N-ω-nitro-L-arginine. T cell proliferation and IFNγ^+^ T cell clone formation partially but significantly recovered in the presence of N-ω-nitro-L-arginine (Fig. 6E). T cell proliferation in allo-MLR, as well as in MLR with Tact-EV or Trest-EV, was not affected by N-ω-nitro-L-arginine (Fig. 6E).

The addition of the membrane-impermeable NO-scavenger carboxy-PTIO did not affect the anti-proliferative activity of dnIKK2-Treg-EV, indicating that the suppressive effect was due to iNOS enzyme within dnIKK2-Treg-EV (Fig. 6F).

### The whole molecular cargo of dnIKK2-Treg-EV is essential for their anti-proliferative effect

**Figure 7:**
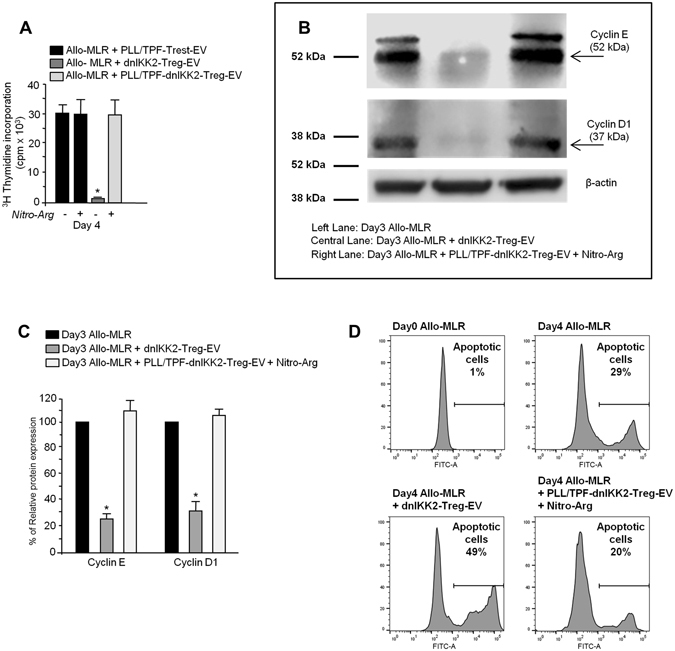
Suppressive effect of dnIKK2-Treg-EV depends on their whole molecular cargo. (**A**) Effect of PLL/TPF-dnIKK2-Treg-EV in combination with NOS inhibition on T cell proliferation. An allogeneic MLR (Allo-MLR, 1 × 10^6^ LW lymph node cells plus 10,000 BN mature DC) was performed in the presence of EV from 20,000 dnIKK2-Trest treated with poly-L-lysine (PLL) and trypaflavine (TPF) (PLL/TPF-dnIKK2-Trest-EV), or 20,000 dnIKK2-Treg (dnIKK2-Treg-EV), or 20,000 dnIKK2-Treg treated with PLL/TPF (PLL/TPF-dnIKK2-Treg-EV) + /− N-ω-nitro-L-arginine (NitroArg). Proliferation was measured by incorporation of ^3^H-Thymidine at day 4 of MLR and expressed as cpm. Results are expressed as mean ± SE of 3 independent experiments. *p < 0.05 vs all groups. (**B,C**) Expression and quantification of Cyclin E and Cyclin D1 in cells from allogeneic MLR performed in the presence of PLL/TPF-dnIKK2-Treg-EV + NOS inhibition. (**B**) Western blot analysis of Cyclin E, Cyclin D1 and β-actin in protein extracts of cells from day 3 MLR with (central lane) or without (left lane) dnIKK2-Treg-EV, or day 3 MLR with PLL/TPF-dnIKK2-Treg-EV in combination with N-ω-nitro-L-arginine (NitroArg) (right lane). 20 μg of total proteins were loaded for each lane. Blots were cropped. Molecular weights are given on the left. One representative experiment of 3 is shown. (**C**) results of densitometric analysis are given, after normalization with β-actin, as % of relative expression, considering Allo-MLR as 100%. Mean ± SD of 3 independent experiments. ^*^p < 0.05 vs all corresponding groups. (**D**) Evaluation of apoptotic cells. FACS analysis of apoptotic cells (by TUNEL assay) at day 0 or day 4 of MLR with or without dnIKK2-Treg-EV, or day 4 of MLR with PLL/TPF-dnIKK2-Treg-EV in combination with N-ω-nitro-L-arginine (NitroArg). The numbers in the histograms give the percentage of apoptotic cells (TUNEL positive cells). One representative experiment of 3 is shown.

To assess whether the whole molecular cargo of dnIKK2-Treg-EV, miRNAs and iNOS, was required for them in order to exert their anti-proliferative effect, MLR experiments were repeated with both EV of dnIKK2-Treg obtained in the presence of PLL/TPF (PLL/TPF-dnIKK2-Treg-EV) and N-ω-nitro-L-arginine. EV of Trest obtained in the presence of PLL/TPF (PLL/TPF-Trest-EV) were used as controls. As shown in Fig. 7A, the combined inhibition of miRNA and iNOS did not affect T cell proliferation in the presence of control Trest-EV while it completely abolished the anti-proliferative effect induced by dnIKK2-Treg-EV.

According to the results of T cell proliferation, the inhibition of both miRNA and iNOS restored the expression of CyclinE and CyclinD1 in T cells exposed to dnIKK2-Treg-EV during MLR, as shown by results of comparable expression levels of Cyclin E and Cyclin D1 in protein extracts of T cells from MLR with PLL/TPF-dnIKK2-Treg-EV and N-ω-nitro-L-arginine and T cells from control MLR (Fig. 7B,C).

Furthermore, the combined inhibition of miRNA and iNOS abolished the pro-apoptotic effect of dnIKK2-Treg-EV. In fact, at the end of MLR performed in the presence of PLL/TPF-dnIKK2-Treg-EV and N-ω-nitro-L-arginine, the percentage of apoptotic T cells was similar to that observed at the end of control MLR and significantly (p < 0.05) lower than that observed at the end of MLR performed in the presence of dnIKK2-Treg-EV (Fig. 7D).

### DnIKK2-Treg-EV prolong kidney allograft survival

**Figure 8:**
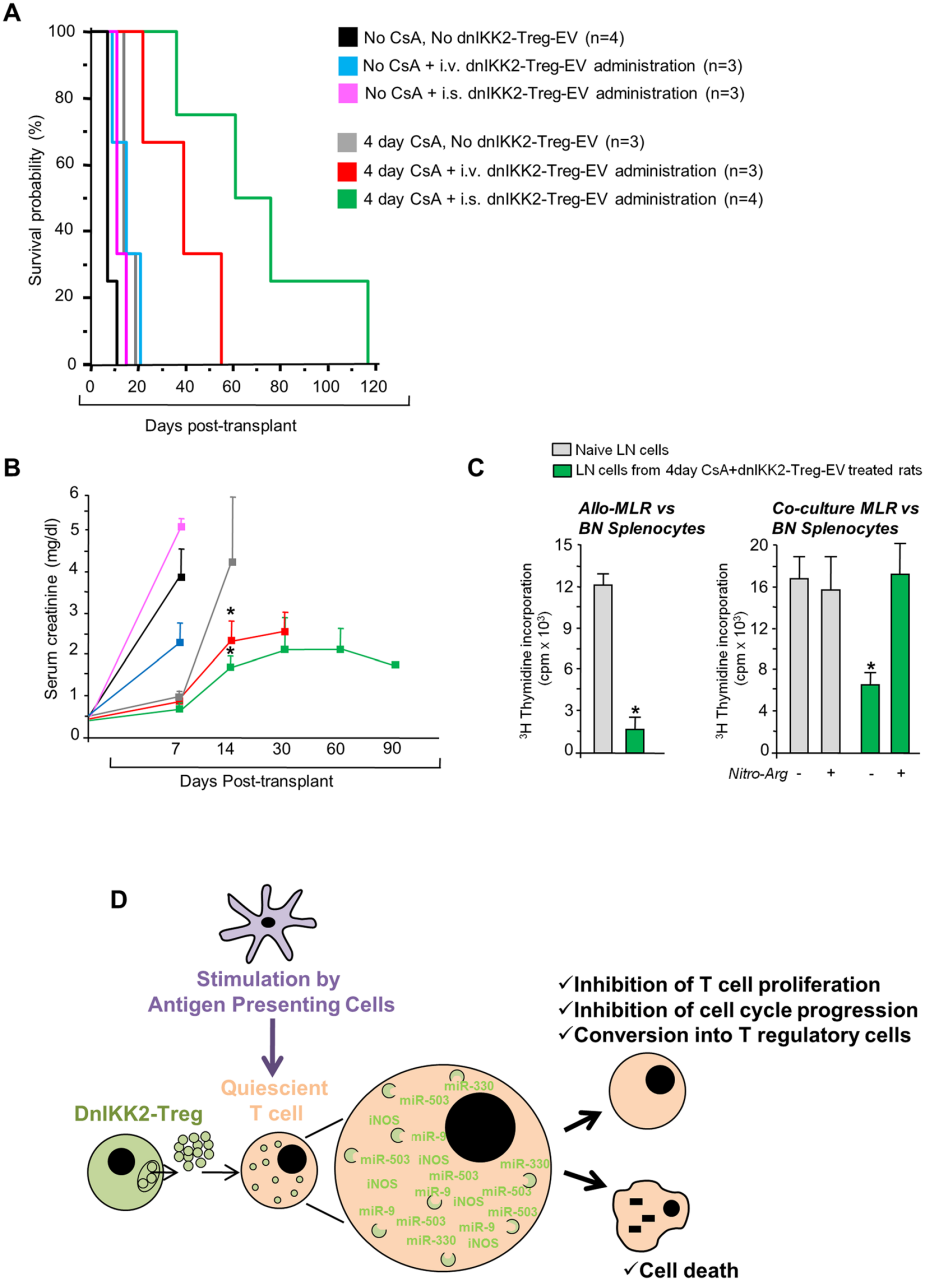
Effect of dnIKK2-Treg-EV on kidney allograft survival. (**A**) Kaplan-Meier analysis of survival. LW recipient rats were treated with EV released by either 100 × 10^6^ (for the intravenous administration route, i.v.) or 25 × 10^6^ (for the intrasplenic administration route, i.s.) dnIKK2-Treg (dnIKK2-Treg-EV). Control groups were i.v. or i.s. treated with vehicle (PBS). The day after i.v. or the day of i.s. dnIKK2-Treg-EV administration, recipient rats were subjected to BN kidney transplantation. Three groups did not receive immunosuppression (n = 3/4 each group), whereas three groups were CsA treated for 4 days after transplantation (n = 3/4 each group). DnIKK2-Treg-EV, administered either i.v. or i.s. and given together with 4-day CsA treatment, significantly prolonged kidney allograft survival (Log-rank test, p < 0.01 vs all the other groups). (**B**) Graft function in kidney allotransplanted rats. Serum creatinine levels in LW rats receiving a BN kidney allograft at 7–90 days post-transplant. Results are mean ± SD. *p < 0.05 vs corresponding group receiving CsA alone. (**C**) Ex-vivo studies. Left panel: a 4-day allogeneic MLR was performed with 1 × 10^6^ irradiated BN splenocytes and 1 × 10^6^ lymph node cells from naïve LW rats (n = 3) or rats treated with 4 day CsA + dnIKK2-Treg-EV, receiving a BN kidney transplant and long-term surviving (>60 days post-transplant, n = 3). Results are mean ± SD. *p < 0.05 vs naïve condition. Right panel: a 4-day co-culture MLR was performed with T cells from naïve LW rats (n = 3) or rats treated with 4 day CsA + dnIKK2-Treg-EV, receiving a BN kidney transplant and long-term surviving (>60 days post-transplant, n = 3) added (at ½ ratio with naïve responder cells) to an Allo-MLR (LW T cells + BN irradiated splenocytes) + /− N-ω-nitro-L-arginine (NitroArg). Proliferation was measured by ^3^H-Thymidine incorporation and expressed as cpm. Results are mean ± SD. *p < 0.05 vs all groups. (**D**) A scheme describing the suggested mechanism of inhibition of T cell proliferation induced by dnIKK2-Treg-EV.

Finally, we verified whether dnIKK2-Treg-EV had immunoregulatory function *in-vivo* in the MHC-mismatched BN (RT1^n^) to LW (RT1^l^) rat model of kidney allotransplantation^29^. The intravenous injection of dnIKK2-Treg-EV in LW recipient animals induced a modest, and not significant, prolongation of allograft survival (15 ± 6 vs 8 ± 2 days in vehicle-treated rats, mean ± SD, Fig. 8A). Death was preceded by a sudden rise of serum creatinine of 2 to 5 mg/dL in both groups (Fig. 8B) indicating that animals died of acute graft rejection.

Since splenic T cells are mainly responsible for early acute allograft rejection^30, 31^, we administered dnIKK2-Treg-EV via intrasplenic inoculation to recipient rats. No significant allograft survival prolongation was observed when recipient rats received dnIKK2-Treg-EV in the spleen (12 ± 2 days post-transplant, n = 3, mean ± SD, Fig. 8A).

To delay acute graft rejection, giving enough time to dnIKK2-Treg-EV to exert their anti-proliferative effect, dnIKK2-Treg-EV were administered together with a four-day Cyclosporine (CsA) treatment. CsA-treated animals showed allograft rejection within 19 days post-transplant (16 ± 3 days post-transplant, n = 3, mean ± SD, Fig. 8A,B). In contrast, when recipient rats were CsA-treated and received dnIKK2-Treg-EV i.v., allograft survival was further prolonged (38 ± 16 days post-transplant, mean ± SD, n = 3, p < 0.01 vs all groups). More importantly, intrasplenic administration of dnIKK2-Treg-EV, together with the short course of CsA, prevented acute rejection and prolonged allograft survival compared with animals that received dnIKK2-Treg-EV injections via i.v. (73 ± 34 days post-transplant, mean ± SD, n = 4, p < 0.01 vs all groups, Fig. 8A), with 75% of recipient rats achieving long-term allograft survival (>60 days post-transplant) and displaying stable renal function (Fig. 8B).

As compared to naïve T cells, T cells obtained from lymph nodes of long-term surviving rats were hyporesponsive vs donor alloantigens (Fig. 8C, left panel). Co-culture experiments documented that T cells from long-term surviving rats suppressed naïve T cell proliferation toward BN alloantigens (Fig. 8C, right panel). Suppressive effect was fully reverted by addition of N-ω-nitro-L-arginine to co-culture MLR, suggesting that iNOS activity might play a crucial role in such regulatory function (Fig. 8C, right panel). By FACS analysis, the percentage of CD25^+^FoxP3^+^ T cells was not different between long-term surviving (6.4 ± 1.9% CD25^+^FoxP3^+^ on CD3^+^CD4^+^ T cells, n = 3) and naïve rats (5.7 ± 1.0% CD25^+^FoxP3^+^ on CD3^+^CD4^+^ T cells, n = 3, Supplementary Fig. 10), confirming *in-vivo* that Treg formed by dnIKK2-Treg-EV were not CD25^+^FoxP3^+^.

## Discussion

In this report we document that dnIKK2-Treg release EV riched in exosomes which potently suppress T cell proliferation, fully mirroring the cell contact-independent immunosuppressive activity of their parent cells.

EV, once they reach the target cells, can be internalized^32^, thereby releasing their content into the cytosol^33, 34^ and modifying or reprogramming the recipient cells^16, 19, 35^. Our finding here that dnIKK2-Treg-EV are taken up by target T cells and that their T cell suppressive activity depends on EV integrity, indicates that the anti-proliferative effect of dnIKK2-Treg-EV relies on the delivery of their cargo into naïve T cells.

In search of mediators of the T cell anti-proliferative effect of dnIKK2-Treg-EV, we focused on microRNAs (miRNAs), based on data that cell-derived EV and exosomes can contain miRNAs which are delivered to another cell, where they can be functional^10, 31, 32, 36, 37^. The analysis of miRNA levels showed that the miRNA cargo of dnIKK2-Treg-EV makes them unique and different from Tact-EV or Trest-EV.

Okoye *et al*. have recently documented that murine CD4^+^CD25^+^Foxp3^+^Treg release miRNA-containing exosomes, that transfer Let-7b, Let-7d and miR-155 into Th1 cells, contributing to suppressing Th1 activation and inflammation in murine colitis^10^. The mechanism seems to involve decreased IFN-γ secretion in Th1 cells following a possible Let-7d-induced Cox-2 inhibition. DnIKK2-Treg-EV, described here, also reduced the formation of IFN-γ^+^ T cell clones, but differed from the Treg-derived-exosomes described by Okoye *et al*. Firstly, the dnIKK2-Treg-EV inhibited T cell activation as effectively as dnIKK2-Treg, consistent with our previous finding that cell-to-cell contact was not essential for their parent cells in order to be suppressive^6^. Second, we did not detect Let-7d in dnIKK2-Treg-EV, but other specific miRNAs. Namely, miR-503, miR-330 and miR-9, which affect the transcription of genes encoding proteins crucial to the regulation of cell cycle progression, were exclusively present or were up-regulated in dnIKK2-Treg-EV compared to Tact-EV and Trest-EV. There is evidence that the over-expression of miR-503 induces a G1 cell cycle arrest in several cell lines by down-regulating genes such as CCNE1 (Cyclin E1), CCND1 (Cyclin D1), CDKN1A (Cip1, p21), CDC25A (Cdc25A phosphatase), CHEK1 (Chk1 kinase) and WEE1 (Wee1 kinase) both at mRNA^38^ and at the protein level^39, 40^. Our findings – that Cyclin E and Cyclin D1 proteins are down-regulated in T cells exposed to dnIKK2-Treg-EV, together with the arrest of T cell cycle progression – confirm that miR-503 has a role in cell cycle regulation in this setting. However, miRNA delivery did not account for 100% of the anti-proliferative effect of dnIKK2-Treg-EV, suggesting the presence of additional anti-proliferative molecules.

In search of additional mediators, we focused on iNOS, which we previously demonstrated was expressed in dnIKK2-Treg^6^. Here we show that the iNOS mRNA and protein were present in dnIKK2-Treg-EV, with the protein appearing more concentrated in the EV compared to the parent Treg. To the best of our knowledge, this is the first report showing the iNOS enzyme within extracellular vesicles. In 1995, a report described a membrane-associated iNOS isoform within 50–80 nm intracellular vesicles, not corresponding to lysosomes or peroxisomes^41^. It is tempting to speculate that the previously documented iNOS-containing intracellular vesicles^41^ and those shown here are the same vesicles, the latter being the extracellular counterparts of the former.

The results of higher iNOS expression in T cells exposed to dnIKK2-Treg-EV, compared to unexposed T cells, would indicate that iNOS mRNA and protein were delivered by the EV into target cells, with intracellular NO-mediated anti-proliferative, cytotoxic and apoptotic effects^42, 43^. In this regard, the over-expression of NOS in human aortic vascular smooth muscle cells was accompanied by lack of cell proliferation and apoptosis^44^. Similarly, in human breast cancer cells exposure to a NO donor caused the arrest of cell cycle progression. This effect was due to a decrease in cyclin D1 synthesis^45^, which was in line with our present data that iNOS-containing EV induced the down-regulation of the cyclin D1 protein and cell cycle arrest in T cells. However, dnIKK2-Treg-EV induced anti-proliferative activity was not entirely due to iNOS, even though it was delivered in an enzymatically active state, as indicated by results showing that N-ω-nitro-L-arginine, but not carboxy-PTIO, a cell-impermeable NO-scavenger^46^, partially recovered the proliferative capacity of dnIKK2-Treg-EV-exposed T cells.

Notably, we found that neither miRNAs nor iNOS accounted by themselves for the anti-proliferative and pro-apoptotic effects of dnIKK2-Treg-EV. Only the combined inhibition of miRNAs and iNOS completely restored proliferation and prevented apoptosis in dnIKK2-Treg-EV exposed T cells, indicating that the whole molecular cargo inhibited T cell alloreactivity. Our data implicate that miRNA and iNOS delivery into T cells by EV blocked cell cycle progression and increased intracellular NO production leading to apoptosis (summarized in Fig. 8D).

DnIKK2-Treg-EV not only inhibited T cell proliferation but also induced target T cells to acquire a regulatory function that is FoxP3 independent. Finding that T cells exposed to dnIKK2-Treg-EV released high amount of IL-10 and suppressed without needing cell-to-cell contact, would suggest that they possess a Tr1-like phenotype^47^ rather than a CD25^+^FoxP3^+^-like phenotype. However, results showing that, at variance with Tr1 cells^48^, T cells exposed to dnIKK2-Treg-EV did not co-express CD49b and LAG3, rule out such possibility. As compared to naïve and activated T cells, the large majority of T cells exposed to dnIKK2-Treg-EV during MLR expressed Tim3, an inhibitory receptor expressed by a unique CD4^+^ Treg population recently described^49, 50^. The exact phenotype of the unconventional induced Treg here reported remains unclear and it is worth of further investigations. However, conversion into Treg exerted by EV derived by dnIKK2-Treg could be reminiscent of the model of infectious tolerance described by Waldmann who first proposed that tolerance can be passed on from one population of lymphocytes to another^51^.

Formation of dnIKK2-Treg-EV-converted Treg could be explained by the link coupling cell cycle regulation and Treg differentiation provided by data that human CD4^+^CD25^−^ T cells treated with anti-CD3/anti-CD28 together with the vasoactive intestinal peptide underwent cell cycle arrest and acquired T cell suppressive activities^52^. Moreover, the down-regulation of cell cycle and Foxo family genes resulted in reprogramming and the conversion of diabetogenic autoreactive T cells to Treg that did not need cell-to-cell contact with target cells^53^, similarly to dnIKK2-Treg-EV-converted Treg here described. Furthermore, modulating cell cycle in T cells plays a role in acquired peripheral tolerance to alloantigens^54^, as Treg from cdk-2-deficient mice display enhanced immunosuppressive function and cdk-2-deficient mice failed to reject a cardiac allograft due to the presence of fewer Th1 and more Foxp3^+^Treg in tolerated grafts compared to rejected grafts from wild type recipients^55^.

Consistent with *in-vitro* data, here we found that treatment with dnIKK2-Treg-EV significantly prolonged kidney allograft survival. Graft survival was more prolonged when dnIKK2-Treg-EV were administered into the recipient spleen, rather than through i.v. injection, according to data documenting that splenic T cells are the main initiators of acute rejection in vascularized transplant models^30, 31^. Notably, prolonging allograft survival required that dnIKK2-Treg-EV be given together with a 4-day CsA treatment, which *per se* did not prevent acute rejection. We hypothesized that the 4-day CsA treatment controlled T cell response until dnIKK2-Treg-EV were fully effective, in line with *in-vitro* results showing that the anti-proliferative effect of dnIKK2-Treg-EV was fully achieved after 4 days of MLR. Finding that T cells harvested from long-term surviving transplanted rats treated with dnIKK2-Treg-EV were hyporesponsive and exerted regulatory function by a mechanism that was dependent on NOS activity, would suggest that dnIKK2-Treg-EV regulated T cell proliferation through similar mechanisms, both *in-vivo* and *in-vitro*. Despite the powerful regulatory function, we recognize that dnIKK2-Treg-EV are deprived of antigen specific effect *in-vitro*. However, it could be tempting to speculate that *in-vivo*, in the context of alloantigen specific T cell stimulation as it occurs in allotransplantation, the suppressive effect of dnIKK2-Treg-EV could result in an antigen specific suppression, as we previously documented in transplanted animals treated with the parent cells dnIKK2-Treg^6^.

Altogether our results show that EV released from dnIKK2-Treg possess a unique molecular cargo, composed by specific miRNAs and iNOS which, once delivered into T cells, inhibited T cell alloreactivity *in-vitro* and *in-vivo* by perturbing cell cycle progression, inducing apoptosis, and converting target T cells into Treg. The use of EV, as compared to their parent cells as therapy to induce immune tolerance in transplantation, could offer some advantages due to the fully cell-free approach, the stable nature of EV after *in-vivo* infusion as well as the easy storage^56^. DnIKK2-Treg-derived EV could be a tool for manipulating the immune system in recipients of solid organ transplants and can open an unanticipated possibility to discover novel potential immunosuppressive molecules to be exploited in the context of allotransplantation.

## Materials and Methods

### Animals and ethics statement

Male inbred Brown Norway (RT1^n^, BN, Charles River, Calco, Italy), DA (RT1^a^, DA, Charles River), Lewis (RT1^l^, LW, Charles River) and Wistar Furth (RT1^u^, WF, Charles River) rats weighing 210 to 250 g (3 months old) were used. These animals differ for class I, class II and non-MHC genes. Procedures involving animals and their care were conducted in conformity with the following laws, regulations and policies governing the care and use of laboratory animals: Italian Governing Law (D.lgs 26/2014; Authorization n.19/2008-A issued March 6, 2008 by Ministry of Health); the NIH Guide for the Care and Use of Laboratory Animals (2011 edition) and EU directives and guidelines (EEC Council Directive 2010/63/UE).

Animal experimental protocols have been approved by our Institutional Committee (IACUC, IRFMN Animal Care and Use Committee) at “IRCCS-Istituto di Ricerche Farmacologiche Mario Negri”, which includes members “ad hoc” for ethical issues. Animals were housed in the Institute’s Animal Care facilities which meet international standards. They were regularly checked by a certified veterinarian who is responsible for health monitoring, animal welfare supervision, experimental protocols and procedures revision.

### Generation of CD4^+^ dnIKK2-regulatory T cells, CD4^+^ activated T cells and CD4^+^ resting T cells

Brown-Norway bone marrow-derived immature or mature DC were obtained as previously described^6, 7^. Briefly, DC were made immature by transfection with adenovirus-encoding dnIKK2 (dnIKK2-DC), whereas DC transfected with empty adenovirus (AdV0-DC) were considered control mature DCs. DnIKK2-DC or AdV0-DC were used as stimulators in a 4-day allogeneic primary mixed leukocyte reaction (MLR) with Lewis (LW) lymph node cells (LN) as responders (1:100 DC/responder ratio). In selected experiments DA lymph node cells were used as responders. At the end of MLR, cells were stained with APC-conjugated anti-rat CD4 (OX35 clone, eBioscience) and CD4^+^ T cells were sorted by FACS (FACSaria, BD, purity: 90–95% on average) to obtain CD4^+^ regulatory T cells (here called *dnIKK2-Treg*) or CD4^+^-activated T cells (named *Tact*), respectively, as previously described^6, 7^. Sorted CD4^+^ dnIKK2-Treg or Tact were then stained by FITC-conjugated anti-CD11c antibody and FACS-analyzed. CD4^+^ sorted cells did not express CD11c marker. Despite this result, suggesting the absence of DC in dnIKK2-Treg preparation, additional experiments were performed to completely rule out the presence of dnIKK2-DC within dnIKK2-Treg. In detail, LN cells from DA rats were stimulated by BN dnIKK2-DC in a 4-day MLR. At the end of the MLR cells were stained with a mouse anti-rat RT1A^a^ antibody (anti-DA MHC class I, clone MN4-91-6, AbDSerotec), followed by FITC-conjugated anti mouse secondary antibody (Invitrogen), and APC-conjugated anti-CD4 antibody. Sorted DA RT1A^a+^CD4^+^ dnIKK2-Treg were 100% negative for CD11c expression. APC and FITC-conjugated control isotype antibodies were used as negative controls. LN cells were cultured alone for 4days and then FACS-sorted to obtain resting CD4^+^ T cells (here called *Trest*).

In order to obtain either dnIKK2-Treg or Trest incapable of generating miRNAs, LN cells were cultured for 4 days, with or without dnIKK2-DC respectively, in the presence of poly-L-lysine (5 μM, poly-L-lysine hydrobromide, MW 4,000–15,000, Sigma Aldrich) and tryplaflavine (8 μM, 3,6-diamino-10-methylacridinium chloride, Sigma Aldrich), two non-cytotoxic small molecules affecting miRNA generation and stability^27, 28^.

DnIKK2-Treg (both from DA and LW rat), or Tact, or Trest were incubated alone (2 × 10^6^/ml) for 18 h in medium supplemented with exosome-free fetal bovine serum (FBS, overnight centrifugation, 100,000 g) to obtain conditioned medium. In selected experiments dnIKK2-Treg were stained with 0.5 μM carboxyfluoresceinsuccinimidyl ester (CFSE) before the 18 h incubation. In additional experiments, conditioned medium from dnIKK2-Treg was subjected to size exclusion filtering (100 kDa, Merck Millipore) or freeze and thaw cycles.

### Preparation of EV from conditioned medium

Extracellular vesicles (EV) were purified from conditioned medium of dnIKK2-Treg or Tact or Trest, as previously reported^57, 58^ and also as suggested by the position paper from the International Society for Extracellular Vesicles^22, 23^. Conditioned medium was centrifuged at 300 g (10 min), 1,200 g (20 min), 10,000 g (30 min), filtered (0.22 μm) and then ultracentrifuged (100,000 g, 1 h, 4 °C, by swinging bucket rotor), washed in PBS and again ultracentrifuged. The pellet from ultracentrifugation of conditioned medium from about 20 × 10^6^ dnIKK2-Treg or Tact or Trest was resuspended in about 200 μl PBS (corresponding to a concentration of EV released from 100,000 cells/μl) and then used *in-vitro* in MLR experiments. In selected experiments, before adding to MLR, EV were subjected to 4–5 cycles of freeze and thaw, or PKH26 stained, or were treated with 10 μg/mL of RNAseA (Ambion Inc.) for 1 h at 37 °C, followed by 10 U/mL of RNase inhibitor, subjected to ultracentrifugation followed by protein content assessment to add a comparable amount of RNAse-treated or untreated EV to MLR. As shown in supplementary Fig. 11, RNAse-treated dnIKK2-Treg-EV were still able to inhibit T cell proliferation toward allogeneic DCs, suggesting that biological function of EV was not associated with RNA being present on their exterior, as also shown by Valadi *et al*.^37^. Since ultracentrifugation could also pellet protein complexes present in the conditioned medium, to evaluate whether possible co-precipitated proteins contributed to the biological function of EV, we fractionated proteins of medium conditioned from dnIKK2-Treg by HPLC and tested each protein fraction. Results that no protein fraction exerted the suppressive effect observed either with dnIKK2-Treg conditioned medium (pre-HPLC) or with dnIKK2-Treg-EV obtained by the same conditioned medium, would rule out the possibility that precipitated proteins might be responsible for the suppressive effect (Supplementary Fig. 12).

In additional experiments, EV from dnIKK2-Treg were fixed in 2% paraformaldehyde for electron microscopy analysis or conjugated to latex-beads for FACS-analysis of surface antigens. Selected EV preparations were used for protein or RNA extraction. In selected experiments PKH26-labeled EV from 200,000 dnIKK2-Treg were incubated with naïve T cells (1 × 10^6^) and 24–48 h later T cells were FACS-analyzed for PKH26 expression.

### FACS analysis

For FACS analysis EV were first PKH26-labeled using a commercially available kit (Sigma-Aldrich) according to the manufacturer’s instructions. The efficiency of labeling of the EV (determined by FACS) was on average 90–100%. PKH26-labeled EV were attached to 4 μm aldehyde/sulfate latex beads (Invitrogen, Carlsbad, CA, USA) by mixing 30 μg EV in a 100 μl volume of beads for 2 h at room temperature. This suspension was diluted to 1 ml with PBS, and the reaction was stopped with 100 mM glycine. EV bound beads were washed in PBS/1% bovine serum albumin (BSA), blocked with 10% FBS, and stained for FACS analysis with fluorescein isothiocyanate (FITC)-conjugated mouse anti-rat CD11c (AbDSerotec, Clone 8A2), or AF647-conjugated mouse anti-rat CD3 (Biolegend, clone 1F4). In selected experiments, EV, purified from conditioned medium from carboxyfluoresceinsuccinimidyl ester (CFSE)-labeled dnIKK2-Treg, were bound to 4 μm aldehyde/sulfate latex beads and FACS-analyzed. FITC or AF647-conjugated control isotype antibodies were used as negative controls.

Antibodies used for FACS analysis of FoxP3^+^ Treg were FITC-anti-rat CD3 (1F4, Biolegend), APC-Cy7-anti-rat CD4 (W3/25, Biolegend), PE-anti-rat CD25 (OX-39, Biolegend) and AF647-anti-mouse/human/rat FoxP3 (150D, Biolegend). Staining was performed on fixed and permeabilized cells from lymph nodes (Permeabilization/Fixation Kit, eBioscience). FACS analysis was performed on viable cells (ViaProbe Cell Viability Solution, BD) by FACS LSR FortessaX-20 (BD).

Antibodies used for FACS analysis of CD49b^+^ LAG3^+^ Treg were hamster anti rat CD49b (HA1/29, BD Pharmingen) followed by FITC-mouse-anti-Armenian and Syrian Hamster IgG cocktail (BD Pharmingen); rabbit anti-rat LAG3 (LifeSpan Biosciences) followed by PE-rat-anti-rabbit Ig (Southern Biotech); APC-anti-rat CD3 (1F4, BD Pharmingen) and APC-Cy7-anti-rat CD4 (W3/25, Biolegend). Antibodies used for FACS analysis of Tim3^+^CD3^+^ cells were FITC-conjugated anti-rat Tim3 (Biorbyt) and APC-conjugated anti-rat CD3 (1F4, BD Pharmingen). FACS analysis was performed by FACS LSR FortessaX-20 (BD).

### Electron microscopy analysis

The EV sample was fixed in 2% paraformaldehyde, and then loaded to copper grids (100 mesh) coated with Formvar. After washing, the grids were contrasted in 2% uranyl acetate, dried, and then examined by transmission electron microscopy (Morgagni 268D; Philips). The identity of the vesicles as exosomes was confirmed by the presence of the tetraspan surface protein CD63 by immunogold labeling of the grids overnight at room temperature with primary antibody for CD63 (dilution 1:100, BD Pharmingen). The grids were then exposed for 1 h to species-specific anti-IgG antibody conjugated to 12 nm colloidal particles.

### RNA isolation and Real-time PCR analysis

Total RNA was isolated using mirVana Isolation Kit (Ambion) according to the manufacturer’s protocol (for dnIKK2-Treg-EV and Trest-EV) or Trizol (for T cells). Contaminating genomic DNA was removed by RNase-free DNase (Promega) for 1 h at 37 °C. The purified RNA (150 ng for dnIKK2-Treg-EV and Trest-EV and 2.5 μg for T cells) was reverse transcribed using VILO SuperScript RT (Invitrogen). No enzyme was added for reverse transcriptase-negative controls.

To amplify cDNA we used SYBR Green PCR Master Mix (Applied Biosystems) according to the manufacturer’s instruction. Primers used to amplify iNOS, Ctla4, Tim3, PD1, Lag3, Itga2 (CD49b) and Gapdh, used as endogenous control, were described in supplementary Table 4. We used the ΔΔCt technique to calculate cDNA content in each sample using as calibrator the cDNA expression in dnIKK2-Trest-EV or in T cells from day0 Allo-MLR, as specified.

### miRNA profiling

The amount of isolated RNA was analysed by NanoDrop ND-1000 (ThermoScientific). EV released from 10^6^ dnIKK2-Treg or Tact or Trest contained 20–30, 10–20, and 10–15 ng of total RNA respectively. The expression of microRNAs (miRNAs) in EV was profiled using stem–loop quantitative RT-PCR (qRT-PCR) miRNA assays on TaqMan low-density array cards (TLDA) (Rodent Array Card A v2.0, Applied Biosystems). The cards containing assays for 375 Rodent mature miRNAs present in the Sanger miRBase v13.0. qRT-PCRs were performed with Megaplex Primers Pool A according to the manufacturer’s instructions. Total RNA (3 µl per sample/card, ~350 ng total RNA) was reverse transcribed using TaqMan miRNA Reverse Transcription Kit (Applied Biosystems) with Megaplex Primers Pool A (Applied Biosystems). The complementary DNA (cDNA) was run on TLDA cards on ViiA7 Real Time PCR System (Applied Biosystems) using the manufacturer’s recommended cycling conditions (50 °C for 2 min, 95 °C for 10 min followed by 40 cycles of 95 °C for 15 s and 60 °C for 1 min, with data collection at the end of each cycle). Threshold cycle (Ct) values > 35 were considered to be below the detection level of the assays, designated ‘undetected’ and excluded from data analysis. Data were analysed using the ΔΔCt method with dnIKK2-Treg-EV as the reference and small nuclear RNA (snRNA) U6 as endogenous control.

### miRNA validation assays

Individual TaqMan miRNA assays (Applied Biosystems) were performed according to the manufacturer’s instructions. Total RNA (10 ng in 5 µl per sample) isolated from EV was converted to cDNA using the microRNA reverse transcriptase Kit (Applied Biosystems) with 3 µl of specific miRNA assay RT primer in a reaction volume of 15 µl. The cDNA was setup in triplicate qRT-PCRs containing 1 µl of specific TaqMan miRNA assay and run on Viia7 Real Time PCR System (Applied Biosystems). Data were analysed using the ΔΔCt method with dnIKK2-Treg-EV as the reference and snRNA U6 as the endogenous control. Specific TaqMan miRNA assays used in this study were: U6 snRNA ID 001973, mmu-miR-293 ID 001794, hsa-miR-330-5p Assay ID 002230, mmu-miR-503 Assay ID 002456, hsa-miR-9 Assay ID 00058, hsa-miR-126-5p Assay ID 000451, rno-miR-207 Assay ID 001315, mmu-miR-297c Assay ID 002480, hsa-miR-484Assay ID 001821.

### Western blot analysis

At the end of ultracentrifugation, EV were resuspended in 0.1 ml lysis buffer (50 mMβ−glicerolphosphate, 2 mM MgCl_2_, 1 mMEGTA, 0.5% Triton X-100, 0.5% NP-40, 1 mM DTT, 1mMpefabloc, 20 mMpepstatin, 20 mMleupeptin, 1000 U/ml aprotinin, Sigma), and then subjected to 4–5 cycles of freeze and thaw. On average, EV released from 10^6^ dnIKK2-Treg contained 2–3 μg of proteins (quantified by the Bradford method, BioRad). Then, proteins were separated on denaturating sodium dodecyl sulfate polyacrylamide gel by electrophoresis, blotted to PVDF membrane, blocked with 5% milk and then incubated with primary antibody (polyclonal goat anti-CD63, T-14, sc25183, Santa Cruz, Santa Cruz, CA, USA; monoclonal mouse anti-tsg101, C-2, sc7964, Santa Cruz; polyclonal goat anti-calnexin, C-20, sc6465, Santa Cruz; polyclonal rabbit anti-iNOS, BD Transduction Laboratories, or polyclonal rabbit anti-actin, aa20–33, Sigma-Aldrich, St Louis, MO, USA). Enhanced chemiluminescence (ECL) Advance (Amersham Biosciences, Piscataway, NJ, USA) was used for detection.

### Evaluation of T-cell proliferation and activation

EV from either dnIKK2-Treg or Tact or Trest were added at day 0 of an allogeneic mixed leukocyte reaction (MLR) carried out with mature BN DC or WF (third party) DC as stimulators and LW lymph node (LN) cells as responders (1:100 ratio). Cultures were maintained in RPMI/FBS medium 20% in 5% CO_2_ in air at 37 °C for 4 days. T cell proliferation was measured at 1, 3 and 4 days by adding 1 micro-Curie (μCi) ^3^H-thymidine for the last 18 h, then the uptake of radioactivity was measured by liquid scintillation counting. Proliferation was expressed as counts per minute (cpm). In selected experiments, dnIKK2-Treg-EV was added 30 min, 1, 2, 4, 6 and 24 h after the addition of the stimulators (either mature DCs or Concanavalin A, ConA, 5 μg/ml) and T cell proliferation was evaluated at the end of the 4-day stimulation. MLR experiments with LN cells obtained from long-term surviving rats were performed using as stimulators irradiated BN splenocytes. In additional experiments, T cell proliferation was evaluated by carboxyfluoresceinsuccinimidyl ester (CFSE) dilution method. Briefly, 10^6^ LN cells were labeled with CFSE (Molecular Probe) before starting MLR. At the end of the 4-day MLR cells were harvested, stained with 7-amino-actinomycin-D (7-AAD) and APC-conjugated anti-rat CD4 (OX35 clone, eBioscience) or phycoerythrin (PE)-conjugated anti-rat CD8 (OX8 clone, eBioscience) or PE-conjugated anti-rat CD3 (G4.18 clone, eBioscience) and analyzed by FACS. In these experiments the percentage of suppression of T cell proliferation was calculated as follows: 100 − (% of cells that proliferated in the presence of dnIKK2-Treg-EV / % of cells that proliferated in the absence of dnIKK2-Treg-EV) × 100.

In selected experiments enzyme-linked immunospot assay (ELISPOT) for Interferon-γ (IFN-γ) was performed after 1, 3 and 4 days of MLR (Becton Dickinson). The resulting spots were counted on a computer-assisted immunospot image analyser (AelvisElispot Scanner system) and expressed as number of spots/100,000 responder cells. Moreover, at the same time points, medium was harvested and Interleukin-10 (IL-10) release was measured by enzyme-linked immunosorbent assay (ELISA), according to the manufacturer’s instructions (Invitrogen, Carlsbad, CA, USA). In selected experiments, MLR were performed in the presence of dnIKK2-Treg-EV and antibodies against Interleukin-12 (IL-12) (0.9 μg/ml, clone 20G101H7, Invitrogen) or IL-10 (1.45 μg/ml, clone 2G101H7, Invitrogen), or in the presence of N-ω-nitro-L-arginine (2 mM, Sigma-Aldrich,)^59–61^ or of carboxy-PTIO (5 μM, Cayman Chemical).

In selected experiments, at the end of MLR carried out with or without dnIKK2-Treg-EV, proteins were extracted by subcellular protein fractionation kit (Thermo Scientific). Proteins were separated on denaturating sodium dodecyl sulfate polyacrylamide gel by electrophoresis, blotted to PVDF membrane, blocked with 5% milk and then incubated with primary antibody (polyclonal rabbit anti-cyclin E, M-20, sc481, Santa Cruz; polyclonal rabbit anti-cyclin D1, M-20, sc718 Santa Cruz, polyclonal rabbit anti-iNOS, BD Transduction Laboratories, or polyclonal rabbit anti-actin, aa20–33, Sigma-Aldrich). Enhanced chemiluminescence (ECL) Advance (Amersham Biosciences, Piscataway, NJ, USA) was used for detection.

For densitometric analysis scanned images of immunoblotted membranes were analyzed by ImageJ

1.48 v software (Wayne Rasband National Institute of Health, USA). β-actin staining was used as loading control. Data are expressed as relative to intensity of the control band considered equal to 1 or 100%, as specified.

### Co-culture MLR

For co-culture experiments, naïve LW LN cells (10^6^) were cultured with allogeneic mature BN (1:100 ratio) for 3 days in the presence or absence of 10,000 cells obtained from primary MLR carried out with dnIKK2-Treg-EV or Tact-EV or Trest-EV. Co-culture experiments with cells from long-term surviving rats were performed with LN cells from long-term surviving rats or naïve LW rats added (at ½ ratio with target responder cells) for 4 days to a MLR performed with LW LN cells cultured with allogeneic irradiated BN splenocytes. T cell proliferation was measured by adding 1 μCi ^3^H-thymidine for the last18 h.

### Analysis of cell cycle, cell division numbers and apoptosis

At the end of MLR performed in the presence of EV from dnIKK2-Treg or Tact or Trest, cell cycle was evaluated by FACS analysis of propidium iodide (PI) incorporation. Briefly, T cells were fixed with 70% ethanol overnight, washed with PBS, incubated with PI (10 μg/mL) and RNase A (100 μg/mL) and FACS-analyzed. Cell division number was measured by FACS evaluation of CFSE labeled cells on vital (7-AAD^−^) cells, followed by analysis with FlowJo software. Apoptosis was evaluated by FACS using the TUNEL method (TUNEL Kit, Boehringer Mannheim, Mannheim, Germany), as previously described^62^. In selected experiments apoptosis was evaluated by AnnexinV/7AAD staining (PE AnnexinV Detection Kit, BD Pharmingen) and FACS analysis.

### Kidney Transplantation

Kidney transplantation was performed as previously described^29^. LW rats were used as recipients and BN rats as donors. To test the immunomodulatory capacity of dnIKK2-Treg-EV, recipient rats were treated with dnIKK2-Treg-EV or vehicle and survival was monitored. Treatments with dnIKK2-Treg-EV comprised intravenous (i.v.) infusion 1 day before transplantation (EV from 100 × 10^6^ dnIKK2-Treg resuspended in 0.5 ml PBS) or inoculation the day of transplantation into the spleen (EV from 25 × 10^6^ dnIKK2-Treg resuspended in 0.2 ml PBS). Rats were given or not cyclosporine (CsA, 5 mg/Kg/day intramuscularly for 4 days; Novartis Farma, Milan, Italy) as specified. Renal function was monitored by evaluating serum creatinine levels using an auto-analyzer at 7, 14, 30 and 60 days post-transplant. Healthy animals that reached post-transplant day 60, were considered long-term surviving animals.

### Statistical analysis

Results were given as mean ± standard error (SE) or standard deviation (SD) as stated. For all parameters, the significance of differences between individual groups was analyzed by one-way analysis of variance (ANOVA). Changes of the various parameters over time were evaluated by ANOVA for repeated measures. All data were analyzed using MedCalc 10.0.1 statistical software. Survival data were analyzed by the log-rank test. Statistical significance was defined as p < 0.05.

## Electronic supplementary material

Supplementary Information
