# Supplementary material for: Extracellular vesicles derived from T regulatory cells suppress T cell proliferation and prolong allograft survival
Source: Sci Rep. 2017 Sep 14;7:11518. doi: 10.1038/s41598-017-08617-3 (PMC5599553; doi:10.1038/s41598-017-08617-3)
Supplement: Supplementary file 1 — Supplementary Information [file 41598_2017_8617_MOESM1_ESM.pdf]

Supplementary Information

Extracellular vesicles derived from regulatory T cells suppress T cell proliferation and prolong allograft survival

Sistiana Aiello[1], Federica Rocchetta[1], Lorena Longaretti[1], Silvia Faravelli[1], Marta Todeschini[1], Linda Cassis[2], Francesca Pezzuto[1], Susanna Tomasoni[1], Nadia Azzollini[1], Marilena Mister[1], Caterina Mele[1], Sara Conti[1], Matteo Breno[1], Giuseppe Remuzzi[1,3,4], Marina Noris[1] and Ariela Benigni[1]

[1]IRCCS - Istituto di Ricerche Farmacologiche Mario Negri, Centro Ricerche Trapianti, "Chiara Cucchi de Alessandri e Gilberto Crespi", Ranica (Bergamo), Italy;

[2]Institut Hospital del Mar d'Investigacions Mèdiques, Barcelona, Spain;

[3]Unit of Nephrology and Dialysis, Azienda Socio-Sanitaria Territoriale (ASST) Papa Giovanni XXIII, Bergamo, Italy;

[4]Department of Biomedical and Clinical Sciences, University of Milan, Milan, Italy.

Corresponding author:

Marina Noris, PhD, Orcid: 0000-0001-7651-5033

IRCCS - Istituto di Ricerche Farmacologiche Mario Negri

Centro Ricerche Trapianti "Chiara Cucchi de Alessandri e Gilberto Crespi"

Via GB Camozzi, 3

24020 Ranica, Italy

Phone n°: 0039 035 4535362

E-mail: marina.noris@marionegri.it

Running title: Immunomodulatory Treg-derived extracellular vesicles

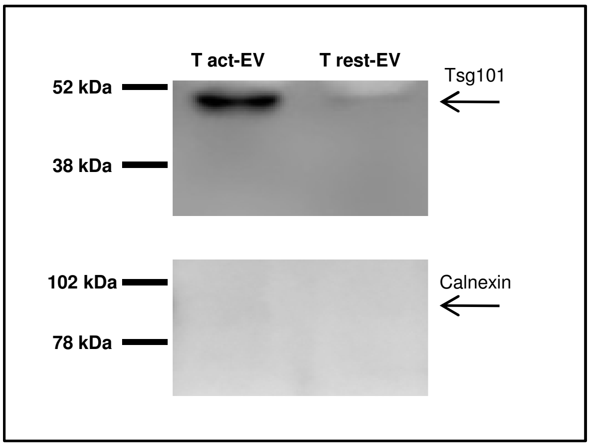

Tsg101 and calnexin protein expression. Tsg101 (upper panel) and calnexin (lower panel) in protein extracts from Tact-EV (left lane) or Trest-EV (right lane) were analyzed by Western blot. For each lane 15µg of total proteins were loaded. Blots were cropped. Molecular weights are given on the left.

Supplementary Figure 1

Numbers of cell divisions in dnIKK2-Treg-EV exposed CD4+ T

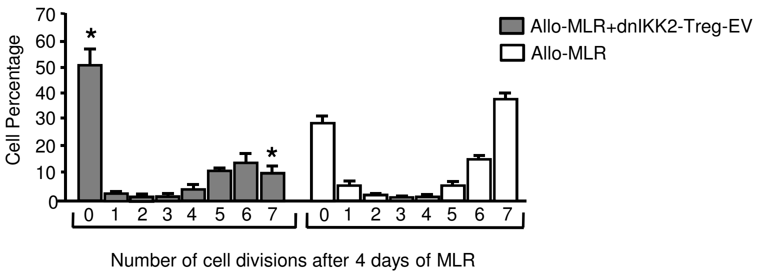

Numbers of cell divisions in dnIKK2-Treg-EV exposed CD8+ T

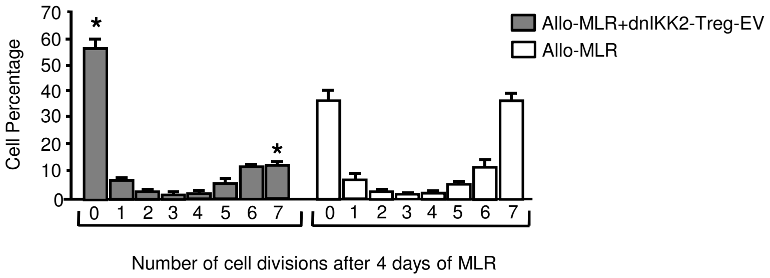

Numbers of cell divisions in dnIKK2-Treg-EV-exposed $CD4^+$ or $CD8^+$ T cells. A 4-day Allo-MLR was performed with or without EV from 20,000 dnIKK2-Treg (dnIKK2-Treg-EV). Cell division number was evaluated by FlowJo software after FACS-analysis of CFSE-labeling on $7\text{-}AAD^-$ $CD4^+$ or $CD8^+$ cells. Results are mean±SD (n=3 independent experiments). *$p<0.05$ vs corresponding Allo-MLR.

Supplementary Figure 2

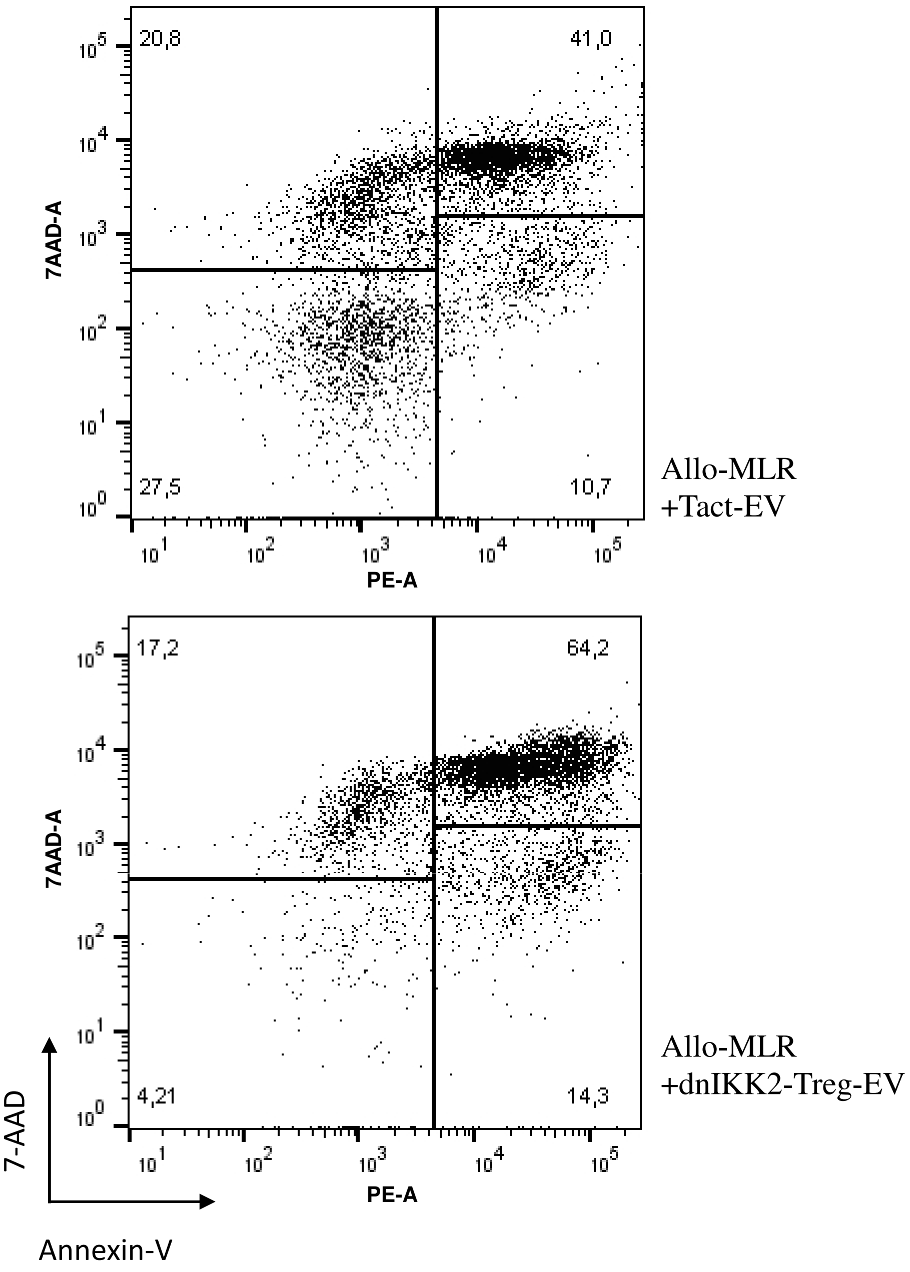

Apoptosis in dnIKK2-Treg-EV-exposed T cells. A 4-day Allo-MLR was performed with dnIKK2-Treg-EV (lower panel) or Tact-EV (upper panel). At day 4, apoptosis was evaluated by AnnexinV/7AAD staining. Percentage of end-stage apoptotic cells (AnnexinV$^+$/7AAD$^+$) is given in upper right panel. Dot plots are representative of two independent and similar experiments.

Supplementary Figure 3

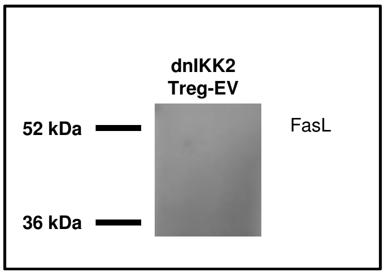

FasL in dnIKK2-Treg-EV. FasL in protein extracts from dnIKK2-Treg-EV was analyzed by Western blot with rabbit anti-FasL antibody (sc-834, Santa Cruz). 20 µg of total proteins was loaded. Blot was cropped. Molecular weights are given on the left.

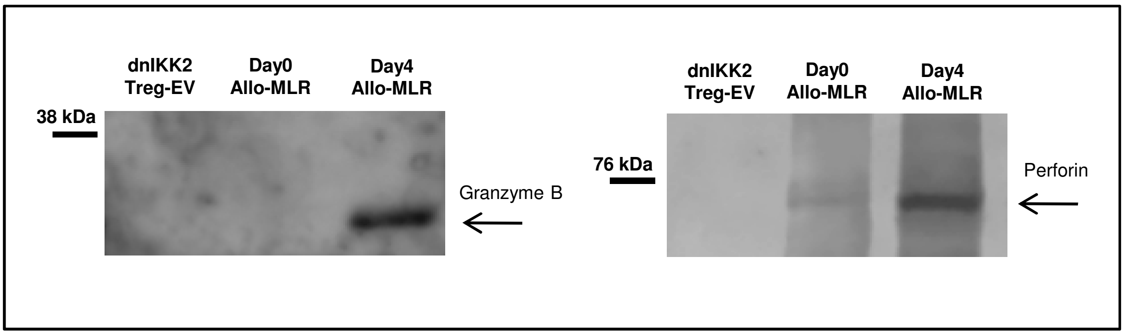

GranzymeB (left panel) and perforin (right panel) expression in dnIKK2-Treg-EV. GranzymeB or perforin in protein extracts from dnIKK2-Treg-EV (left lane) or from day4 Allo-MLR (as positive ctr, right lane) or day0 Allo-MLR (central lane) was analyzed by Western blot with goat anti-granzymeB antibody (sc-1968, Santa Cruz) or with goat anti-perforin antibody (sc-7417, Santa Cruz). For each lane 20 µg of total proteins were loaded. Blots were cropped. Molecular weights are given on the left.

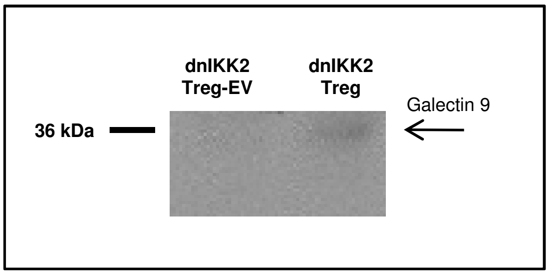

Galectin 9 expression in dnIKK2-Treg-EV. Galectin 9 in protein extracts from dnIKK2-Treg-EV (left lane) or dnIKK2-Treg (right lane) was analyzed by Western blot with goat anti-Gal9 antibody (sc19294, Santa Cruz). For each lane 20 µg of total proteins were loaded. Blot was cropped. Molecular weights are given on the left.

Supplementary Figure 4

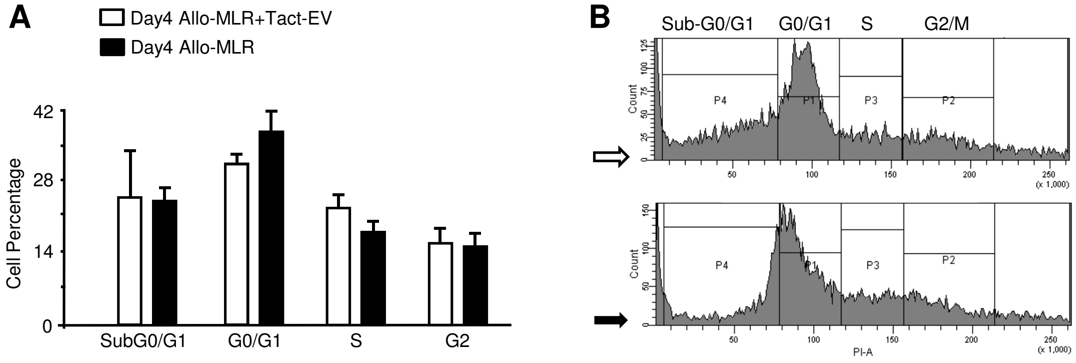

Cell cycle distribution in ctr Allo-MLR with or without Tact-EV
A 4-day Allo-MLR ($1x10^6$ LW lymph-node cells+10,000 BN mature DC) was performed with or without EV from 20,000 activated T cells (Tact-EV). *Panel A*: percentages of T cells in sub-G0/G1, G0/G1, S and G2/M phases. Results are mean±SD (n=4 independent experiments). *Panel B*: Representative FACS histograms of PI staining and gates at day 4 of MLR (upper histogram: allo-MLR in the presence of Tact-EV; lower histogram: allo-MLR in the absence of Tact-EV).

Supplementary Figure 5

A

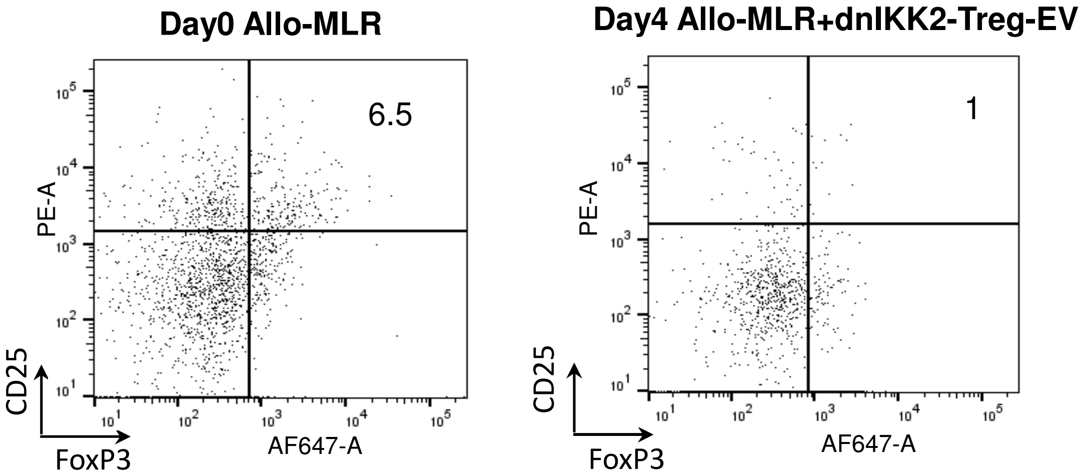

Two representative dot plots showing FACS analysis of $CD25^+Foxp3^+$ cells on $CD3^+CD4^+$ T cells at Day0 Allo-MLR (left dot plot) and at the end of Allo-MLR performed in the presence of dnIKK2-Treg-EV (Day4 Allo-MLR+dnIKK2-Treg-EV, right dot plot). Percentage of $CD25^+Foxp3^+$ cells is given in the upper right quadrant.

B

CD49b and LAG3 mRNA expression (Arbitrary Unit)

	**CD49b**	**LAG3**
**Day0 Allo-MLR**	1	1
**Day4 Allo-MLR**	0.424±0.07	3.59±1.83
**Day4 Allo-MLR+ dnIKK2-Treg-EV**	0.401±0.07	2.74±1.23

CD49b and LAG3 mRNA analysis, by real-time PCR, in T cells exposed (Day4 Allo-MLR+dnIKK2-Treg-EV) or not (Day4 Allo-MLR) to dnIKK2-Treg-EV.The cDNA content was calculated by ΔΔCt technique, using as calibrator the cDNA expression in T cells from Day0 Allo-MLR. Results are expressed as arbitrary units (AUs), mean±SD (n=3 independent experiments).

C

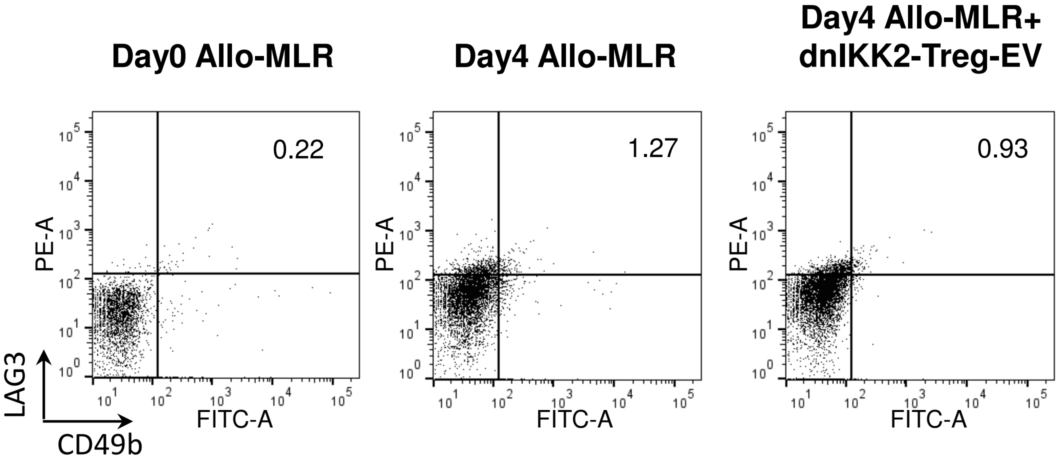

Three representative dot plots showing FACS analysis of $CD49b^+LAG3^+$ cells on $CD3^+CD4^+$ T cells exposed (Day4 Allo-MLR+dnIKK2-Treg-EV, right dot plot) or not (Day4 Allo-MLR, central dot plot) to dnIKK2-Treg-EV, and at Day0 Allo-MLR (left dot plot). Percentage of $CD49b^+LAG3^+$ cells is given in the upper right quadrant.

Supplemetary Figure 6

A **PD-1, CTLA-4, Tim3 mRNA expression (Arbitrary Unit)**

	PD-1	CTLA-4	Tim3
**Day0 Allo-MLR**	1	1	1
**Day4 Allo-MLR**	0.36±0.22	1.87±1.11	3.98±2.73
**Day4 Allo-MLR+ dnIKK2-Treg-EV**	0.37±0.07	3.55±1.58	8.14±1.31*

PD-1, CTLA-4 and Tim3 mRNA analysis, by real-time PCR, in T cells exposed (Day4 Allo-MLR+dnIKK2-Treg-EV) or not (Day4 Allo-MLR) to dnIKK2-Treg-EV.The cDNA content was calculated by ΔΔCt technique, using as calibrator the cDNA expression in T cells from Day0 Allo-MLR. Results are expressed as arbitrary units (AUs), mean±SD (n=3 independent experiments). *$p<0.05$ vs Day4 Allo-MLR.

B **Tim3 expression on T cells**

	Day0 Allo-MLR	Day4 Allo-MLR	Day4 Allo-MLR+ dnIKK2-Treg-EV
**$Tim3^+CD3^+$ T cells (% on $CD3^+$ T cells)**	5.4±3.9	37.6±16.1	74.8±3.6*

Percentage of $Tim3^+$ cells on $CD3^+$ T cells, by FACS analysis, at Day0 Allo-MLR or in T cells exposed (Day4 Allo-MLR+dnIKK2-Treg-EV) or not (Day4 Allo-MLR) to dnIKK2-Treg-EV. Results are mean±SD (n=3 independent experiments). *$p<0.05$ vs Day4 Allo-MLR.

C

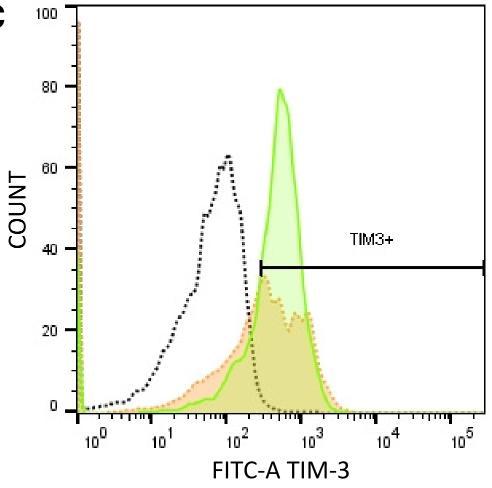

Representative histograms showing FACS analysis of Tim3 expression on $CD3^+$ T cells at Day0 Allo-MLR (dotted histogram), or on cells exposed (Day4 Allo-MLR+dnIKK2-Treg-EV, green histogram) or not (Day4 Allo-MLR, orange histogram) to dnIKK2-Treg-EV.

Supplemetary Figure 7

Genes of cell cycle regulation targeted by miR-503, miR-330 and miR-9

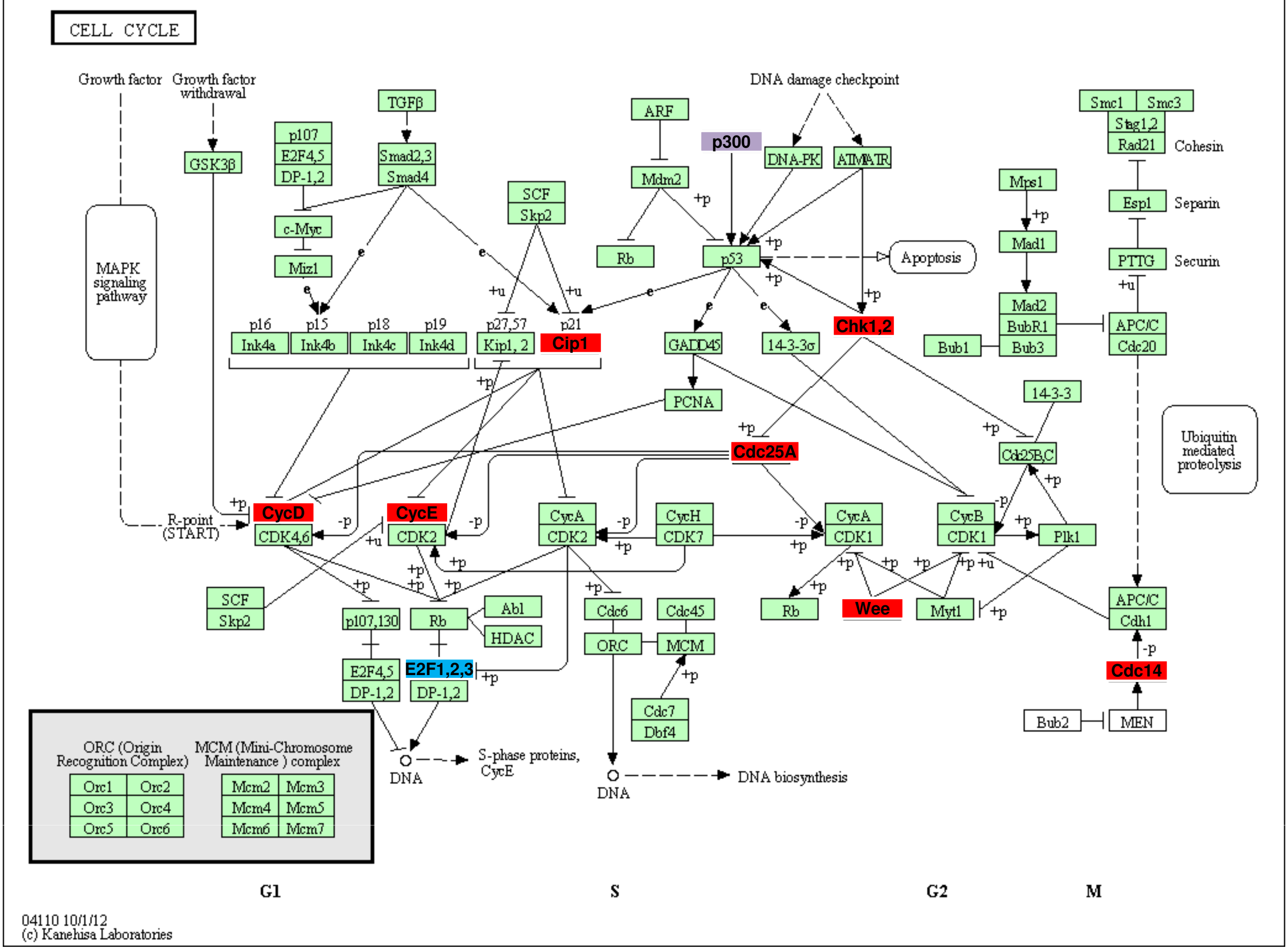

Genes targeted by miR-503:

- **CCND1** (corresponding protein: CycD1)
- **CCNE1** (corresponding protein: CycE1)
- **CCNE2** (corresponding protein: CycE2)
- **CDKN1A** (corresponding protein: Cip1, p21)
- **CDC25A** (corresponding protein: Cdc25A phosphatase)
- **CHEK1** (corresponding protein: Chk1 kinase)
- **WEE1** (corresponding protein: Wee1 kinase)
- **CDC14A** (corresponding protein: Cdc14A phosphatase)

Gene targeted by miR-9:

- **EP300** (corresponding protein: p300 histone acetyltransferase)

Gene targeted by miR-330:

- **E2F1** (corresponding protein: E2F transcription factor 1)

A scheme, modified from KEGG Pathway (www.genome.jp/kegg/pathway.html), showing the genes of cell cycle regulation targeted by miR503, or miR-330 or miR-9, as resulting from the analysis performed with miRPath software considering only gene-miRNA interactions that had been validated in literature in humans.

•Kanehisa, M., Goto, S., Sato, Y., Kawashima, M., Furumichi, M., and Tanabe, M.; Data, information, knowledge and principle: back to metabolism in KEGG. Nucleic Acids Res. 42, D199–D205 (2014).
•Kanehisa, M. and Goto, S.; KEGG: Kyoto Encyclopedia of Genes and Genomes. Nucleic Acids Res. 28, 27-30 (2000).

Supplementary Figure 8

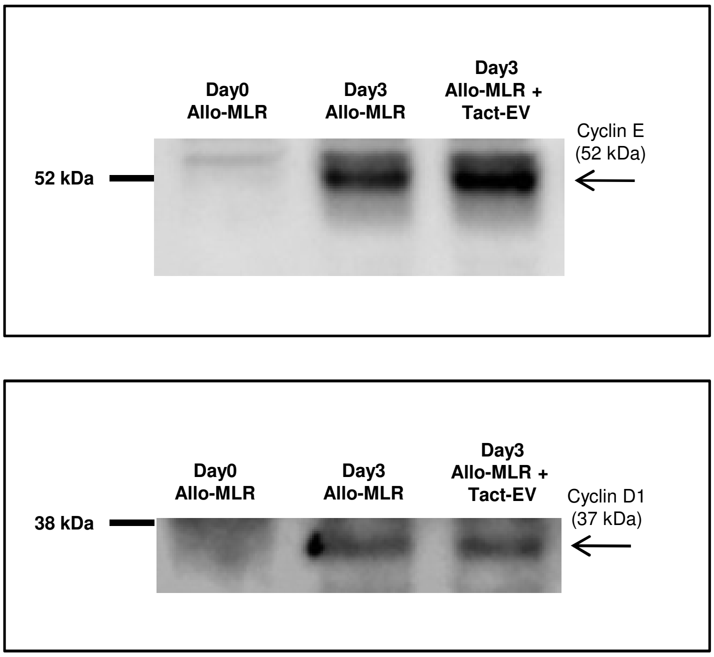

Cyclin E (upper panel) and Cyclin D1 (lower panel) expression. Cyclin E and Cyclin D1 expressions were evaluated in protein extracts of cells from day 0 MLR (left lane) or day 3 MLR with (right lane) or without (central lane) Tact-EV. For each lane 19µg of total proteins were loaded. Blots were cropped. Molecular weights are given on the left.

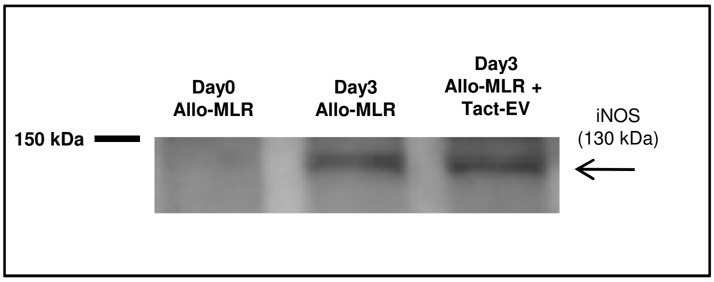

iNOS protein expression. iNOS protein expression was evaluated in protein extracts of cells from day 0 MLR (left lane) or day 3 MLR with (right lane) or without (central lane) Tact-EV. For each lane 19µg of total proteins were loaded. Blots were cropped. Molecular weights are given on the left.

Supplementary Figure 9

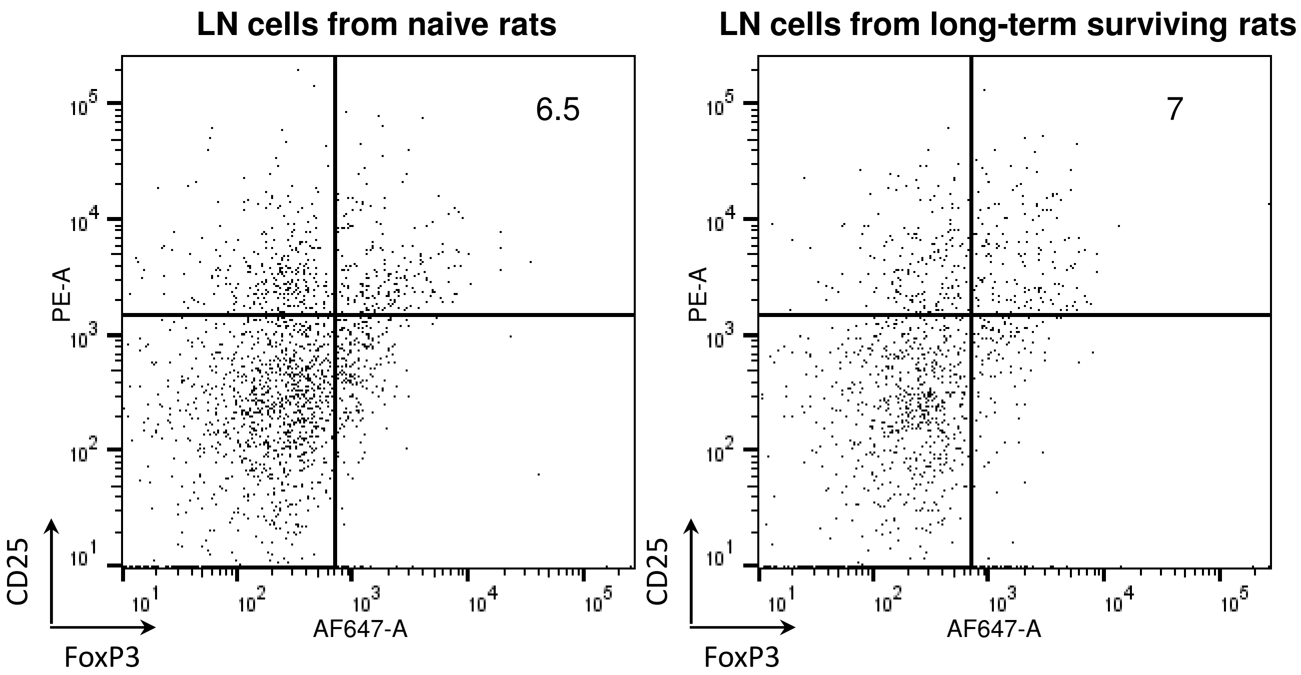

Two representative dot plots showing FACS analysis of CD25^{+}Foxp3^{+} cells on CD3^{+}CD4^{+} T cells from lymph nodes of LW naive rats (left dot plot) or transplanted rats treated with dnIKK2-Treg-EV+4day CsA and long-term surviving (right dot plot). Percentage of CD25^{+}Foxp3^{+} cells is given in the upper right quadrant.

Supplemetary Figure 10

Effect of RNAse treatment on anti-proliferative activity of dnIKK2-Treg-EV

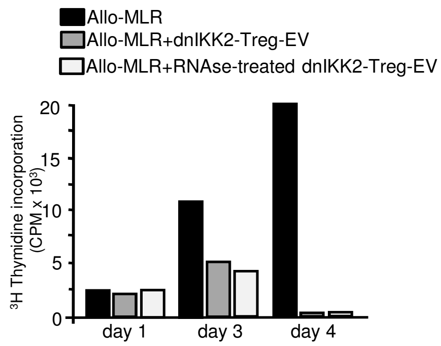

A naive allogeneic MLR (Allo-MLR, $1x10^6$ LW lymph node cells plus 10,000 BN mature DCs) was performed in the presence or absence of EV from 20,000 dnIKK2-Treg (dnIKK2-Treg-EV) treated or not with RNAse and thereafter corrected for protein content. Proliferation was measured by incorporation of ^{3}H-Thymidine at day1, 3 and 4 of MLR and expressed as cpm. Results are mean of 2 independent experiments.

Supplementary Figure 11

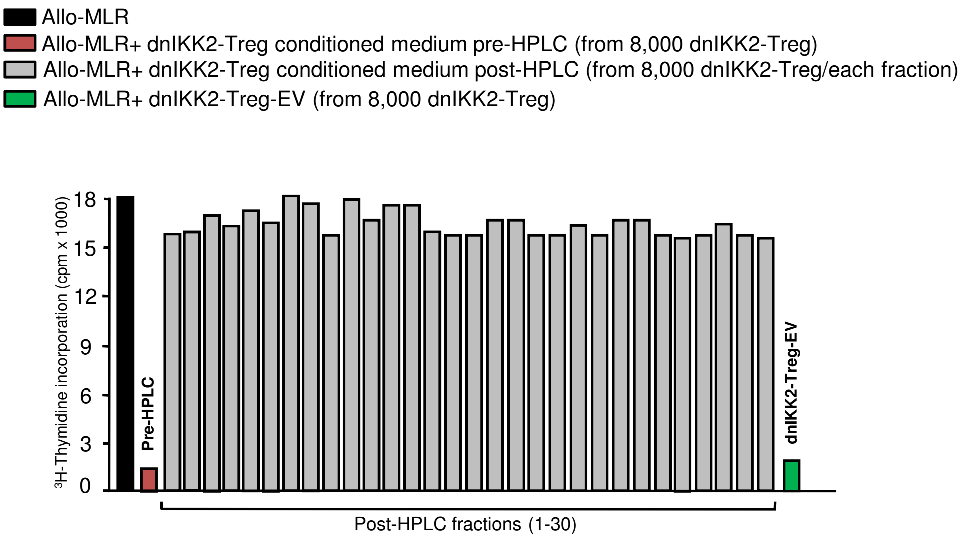

HPLC fractioning of proteins in conditioned medium from dnIKK2-Treg. The chromatographic separation was carried out using a gel filtration Zorbax GF-250 250 x 9.4 mm - 4µm column (CPS Analitica Milan, Italy). The eluate was monitored at 214nm wavelength and up to 30 fractions of 0.5 mL were collected for subsequent analysis. A 4-day allogeneic MLR ($1x10^6$ LW lymph node cells plus 10,000 BN mature DCs) was performed in the presence or absence of conditioned medium pre-HPLC (from 8,000 dnIKK2-Treg), conditioned medium subjected to HPLC fractioning (1-30 post-HPLC fractions, from 8,000 dnIKK2-Treg/each fraction), or conditioned medium subjected to ultracentrifugation (EV from 8,000 dnIKK2-Treg). Proliferation was measured by incorporation of ^{3}H-Thymidine and expressed as cpm.

Supplemetary Figure 12

Supplementary Table 1: miRNA exclusively expressed in dnIKK2-Treg-EV

	dnIKK2-Treg EV	Trest EV	Tact EV
**mmu-miR-677**	**>25<30**	**nd**	**nd**
**mmu-miR-330**	**>25<30**	**nd**	**nd**
**mmu-miR-293**	**>25<30**	**nd**	**nd**
**mmu-miR-9**	**>25<30**	**nd**	**nd**
**mmu-miR-126-5p**	**>25<30**	**nd**	**nd**
**mmu-miR-503**	**>25<30**	**nd**	**nd**
**rno-miR-207**	**>25<30**	**nd**	**nd**
**mmu-miR-297c**	**>25<30**	**nd**	**nd**
**mmu-miR-484**	**>25<30**	**nd**	**nd**
mmu-miR-486	>30<35	nd	nd
mmu-miR-687	>30<35	nd	nd
mmu-miR-130b	>30<35	nd	nd
mmu-miR-467e	>30<35	nd	nd
mmu-miR-320	>30<35	nd	nd
mmu-miR-667	>30<35	nd	nd
mmu-miR-433	>30<35	nd	nd
mmu-miR-467c	>30<35	nd	nd
mmu-miR-139-5p	>30<35	nd	nd
mmu-miR-132	>30<35	nd	nd
mmu-miR-26a	>30<35	nd	nd
mmu-miR-93	>30<35	nd	nd
mmu-miR-494	>30<35	nd	nd
rno-miR-347	>30<35	nd	nd
mmu-miR-743a	>30<35	nd	nd
mmu-miR-186	>30<35	nd	nd
mmu-miR-324-3p	>30<35	nd	nd
mmu-miR-146b	>30<35	nd	nd
mmu-miR-24	>30<35	nd	nd
mmu-miR-20a	>30<35	nd	nd
mmu-miR-199a-3p	>30<35	nd	nd
mmu-miR-126-3p	>30<35	nd	nd
rno-miR-327	>30<35	nd	nd
mmu-miR-331-3p	>30<35	nd	nd
mmu-miR-21	>30<35	nd	nd
mmu-miR-187	>30<35	nd	nd
mmu-miR-665	>30<35	nd	nd
mmu-miR-744	>30<35	nd	nd
mmu-miR-223	>30<35	nd	nd
mmu-miR-491	>30<35	nd	nd
mmu-miR-328	>30<35	nd	nd
mmu-miR-370	>30<35	nd	nd
mmu-miR-331-5p	>30<35	nd	nd
mmu-miR-145	>30<35	nd	nd
mmu-miR-193	>30<35	nd	nd
mmu-miR-188-5p	>30<35	nd	nd
mmu-miR-30a	>30<35	nd	nd
mmu-miR-let-7g	>30<35	nd	nd
mmu-miR-30c	>30<35	nd	nd
mmu-miR-221	>30<35	nd	nd
mmu-miR-210	>30<35	nd	nd
mmu-miR-195	>30<35	nd	nd
mmu-miR-197	>30<35	nd	nd
mmu-miR-680	>30<35	nd	nd
mmu-miR-19a	>30<35	nd	nd
mmu-miR-20b	>30<35	nd	nd
mmu-miR-30d	>30<35	nd	nd

Results are given as Ct values
nd: not detectable

Supplementary Table 2: miRNA not exclusively expressed in dnIKK2-Treg-EV

	dnIKK2-Treg EV	Trest EV	Tact EV
mmu-miR-135b	1	nd	102.97
mmu-miR-150	1	8.94	0.22
mmu-miR-191	1	5.94	nd
mmu-miR-155	1	8.94	1.56
mmu-miR-146a	1	7.06	nd
mmu-miR-16	1	7.84	nd
mmu-miR-685	1	12.3	0.35
mmu-miR-31	1	44.32	nd
mmu-miR-342-3p	1	21.26	nd
mmu-miR-106a	1	6.23	nd
mmu-miR-17	1	5.66	nd
mmu-miR-19b	1	18.9	nd
mmu-miR-92a	1	18.13	nd
mmu-miR-222	1	14.32	4.44
mmu-miR-29a	1	120.26	nd
mmu-miR-15b	1	19.7	91.65
mmu-miR-142-3p	1	nd	17.25

Results are given as arbitrary unit
We used the $\Delta\Delta$Ct technique to calculate cDNA content in each sample using the cDNA expression in dnIKK2-Treg-EV as calibrator.

Supplementary Table 3: results of miRPath software analysis for miRNAs expressed in dnIKK2-Treg-EV using only validated gene-miRNA interactions

#KEGG pathway	p-value	#genes	#miRNAs
Glycosaminoglycan biosynthesis chondroitin sulfate (hsa00532)	5.63^-9	1	1 (miR-9)
Pathways in cancer (hsa05200)	3.33^-7	14	3 (miR-9, miR-330, miR-503)
HTLV-I infection (hsa05166)	2.22^-6	14	3 (miR-9, miR-330, miR-503)
Prostate cancer (hsa05215)	3.43^-6	8	3 (miR-9, miR-330, miR-503)
p53 signaling pathway (hsa04115)	5.94^-6	7	2 (miR-9, miR-503)
Hepatitis B (hsa05161)	1^-5	8	3 (miR-9, miR-330, miR-503)
Viral carcinogenesis (hsa05203)	1.23^-5	9	2 (miR-9, miR-503)
**Cell cycle (hsa04110)**	**2.24^-5**	**10**	**3 (miR-9, miR-330, miR-503)**
Bladder cancer (hsa05219)	3.24^-5	5	3 (miR-9, miR-330, miR-503)
Sphingolipid metabolism (hsa00600)	0.002098815	3	1 (miR-9)
Small cell lung cancer (hsa05222)	0.002458709	5	3 (miR-9, miR-330, miR-503)
Gastric acid secretion (hsa04971)	0.004342629	5	1 (miR-9)
Melanogenesis (hsa04916)	0.005278876	6	1 (miR-9)
Melanoma (hsa05218)	0.01445503	4	3 (miR-9, miR-330, miR-503)
Pancreatic cancer (hsa05212)	0.03174977	4	3 (miR-9, miR-330, miR-503)
Glioma (hsa05214)	0.04640901	4	3 (miR-9, miR-330, miR-503)

Supplementary Table 4: Primer sequences used for gene expression analysis

Primer sequences used for SYBR GREEN Real Time RT-PCR

	**Forward**	**Reverse**
iNOS	GGAGAAAACCCCAGGTGCTAT	CATTCTGRGCAGTCCCAGTGA
Ctla4	GCCCAGATTCAGACTTCCTCC	TCTTTTCTTTAGCGTCCTGTTCAA
Tim3	GCTGACTCTGGGACCTACTG	TGGGATGACTTTGGCTGGTTC
PD1	ATGTCAGAGGCCAGAGAAGC	GTCCCTGAAAGTCCAGCTCC
Lag3	ACCTTTTTCTGAACTCCCTTGC	GACACAACTGGAGCTTCCCA
Itga2 (CD49b)	AGGTTTGGCATAGCGGTTCT	ACCTCTCCGTTGGAGTGCTA
Gapdh	TCATCCCTGCATCCACTGGT	CTGGGATGACCTTGCCCAC
